# Nanoscale Decoupling of Carrier–Phonon Transport in Carbon Nanotube–Halide Perovskite Heterostructures

**DOI:** 10.1002/advs.202507589

**Published:** 2025-09-03

**Authors:** Md Azimul Haque, Taocheng Yu, Hitarth Choubisa, Luis Huerta Hernandez, Yuan Zhou, Alessandro Genovese, Bambar Davaasuren, Craig Combe, Hanying Li, Joseph M. Luther, Jeffrey L. Blackburn, Edward H. Sargent, Wee‐Liat Ong, Derya Baran

**Affiliations:** ^1^ Material Science and Engineering Program (MSE) Physical Sciences and Engineering Division (PSE) King Abdullah University of Science and Technology (KAUST) Thuwal 23955‐6900 Kingdom of Saudi Arabia; ^2^ National Renewable Energy Laboratory Golden CO 80401 USA; ^3^ ZJU‐UIUC Institute College of Energy Engineering Zhejiang University Haining Jiaxing Zhejiang 314400 China; ^4^ Department of Electrical and Computer Engineering University of Toronto 35 St George Street Toronto ON M5S 1A4 Canada; ^5^ Corelabs King Abdullah University of Science and Technology (KAUST) Thuwal 23955‐6900 Saudi Arabia; ^6^ MOE Key Laboratory of Macromolecular Synthesis and Functionalization International Research Center for X Polymers Department of Polymer Science and Engineering Zhejiang University Hangzhou 310027 China; ^7^ State Key Laboratory of Clean Energy Utilization Zhejiang University Hangzhou Zhejiang 310027 China

**Keywords:** halide perovskite, heterostructure, molecular dynamics, phonon transport, thermoelectrics

## Abstract

In conventional semiconductors, electrical and thermal conductivity are typically coupled, posing a challenge in optimizing both simultaneously. Overcoming this inherent trade‐off enables strategies for advancing electronic applications. Herein, a strategy is demonstrated to decouple electrical and thermal conductivity trade‐off by creating heterostructures of highly conductive single‐walled carbon nanotubes (SWCNTs) coated with low conductivity hybrid perovskites. Coating SWCNTs with methylammonium lead iodide perovskite results in an enhancement in electrical conductivity (408–1266 S cm^−1^) due to p‐type doping followed by a threefold decrease of the in‐plane thermal conductivity (3.3–1 W m^−1^ K^−1^), compared to pristine SWCNTs. Molecular dynamics simulations uncover phonon boundary scattering at the SWCNT/perovskite interface as well as localization of methylammonium‐related and softening of the Pb─I‐related phonon modes in methylammonium lead iodide perovskite decreasing the thermal conductivity.

## Graphical abstract

Heterostructures of highly conductive single‐walled carbon nanotubes with low conductivity hybrid perovskites break the trade‐off between electrical and thermal conductivity. This design boosts electrical conductivity while significantly lowering thermal conductivity.

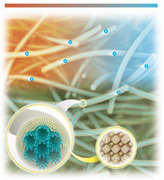

## 1 Introduction

Electrical and thermal conductivity are key parameters that dictate device performance across diverse applications, spanning microelectronics, energy conversion, and sensing.^[^ ^1^, ^2^, ^3^ ^]^ The intricate balance between the two holds paramount significance in the realms of semiconductor devices, electronic packaging, transparent oxide films, flexible electronics, and thermoelectrics.^[^ ^4^, ^5^, ^6^, ^7^ ^]^ Applications such as printed circuits require both high electrical and thermal conductivity for efficient signal transmission and heat dissipation, respectively, whereas thermoelectric devices need high electrical, but low thermal conductivity. A material's thermal conductivity consists of both lattice (phonon) and electrical (electron) components, the latter of which implies a close correlation of both electrical and thermal conductivity to the carrier density in most materials. Independent optimization and decoupling of electrical and thermal conductivity can foster the realization of advanced electronics and has been a focal point of research for several decades.

Single‐walled carbon nanotubes (SWCNTs) have attracted significant interest for advanced electronic devices, owing to their exceptional electrical conductivity and their tunable Fermi level, majority carrier type and density.^[^ ^8^, ^9^, ^10^ ^]^ Heterostructures of SWCNTs with polymers and additives have exhibited promising performance in energy conversion devices.^[^ ^11^, ^12^, ^13^ ^]^ Notably, since the thermal conductivity of SWCNTs is dominated by a relatively high lattice contribution, composites can exploit heterointerfaces to reduce thermal conductivity via phonon scattering without adversely affecting electrical conductivity.^[^ ^14^, ^15^, ^16^ ^]^ Such heterostructures can decouple electrical and thermal transport. Recently, SWCNTs have been employed in concert with halide perovskites as charge extraction layers and in perovskite matrices to form phototransistors and photodetectors.^[^ ^17^, ^18^, ^19^, ^20^, ^21^, ^22^, ^23^ ^]^ These heterostructures have demonstrated broadly tunable (opto)electronic properties and responses.^[^ ^24^, ^25^, ^26^ ^]^ While lead‐halide perovskites feature reasonable charge carrier mobility and electrical conductivity (but several orders of magnitude lower than SWCNTs), their ultralow thermal conductivity impedes heat transport, a combination commonly touted as a “phonon glass, electron crystal.”^[^ ^27^ ^]^ With these complementary properties in mind, we hypothesized that heterostructures combining lead‐halide perovskites with high conductivity SWCNTs could offer a synergistic platform for decoupling electrical and thermal conductivity. In particular, the significant phonon spectra mismatch between SWCNTs and perovskites offers the prospect of independently tuning the electrical and thermal conductivity.

In this study, we coated high conductivity SWCNTs with methylammonium lead iodide (MAPbI_3_) perovskite via solution processing, creating composite heterostructures with decoupled electrical and thermal transport. We observe ground‐state charge transfer across the SWCNT/MAPbI_3_ interface resulting in large p‐type electrical conductivity (1266 S cm^−1^ at 103 °C) in the SWCNT phase. The ultralow thermal conductivity of the MAPbI_3_, coupled with its phonon spectra mismatch with SWCNTs, leads to threefold reduction in the heterostructure thermal conductivity, compared to pristine SWCNT films. Molecular dynamics simulations uncover two competing thermal conductivity mechanisms induced by the MAPbI_3_ coating: twofold increase in thermal conductance of a coated inter‐SWCNT junction (intertube), counteracted by two orders of magnitude drop in thermal conductance of a SWCNT–MAPbI_3_ heterostructure (intratube). Furthermore, strong phonon boundary scattering at the SWCNT/MAPbI_3_ interface is observed, explaining the substantial drop in thermal conductivity. The localization of the MA‐molecule‐related phonon modes and softening of the inorganic‐cage‐related phonon modes reduce the thermal conductivity of MAPbI_3_ in the heterostructure by more than 30%. The MAPbI_3_ coating plays a dual role, by contributing electrically conductive holes to the composite system while simultaneously inducing interfacial phonon scattering. To show the potential of decoupling electrical and thermal conductivity, proof‐of‐concept SWCNT/MAPbI_3_ hybrid films exhibit an improved thermoelectric figure of merit, *ZT* of 0.07 at 103 °C, surpassing the *ZT* values of pristine SWCNTs and pure MAPbI_3_. Furthermore, these hybrid films demonstrate excellent shelf‐life stability of over 1000 h in ambient conditions. This integrated approach presents a promising avenue for thin‐film electronic devices, such as resistive switching, thermal data storage, actuators, and energy converters based on perovskite/SWCNT heterostructures, where electrical and thermal conductivity can be finely tuned and ultimately decoupled.

## 2 Results and Discussion

### 2.1 Halide Perovskite–SWCNT Heterostructures

**Figure 1:**
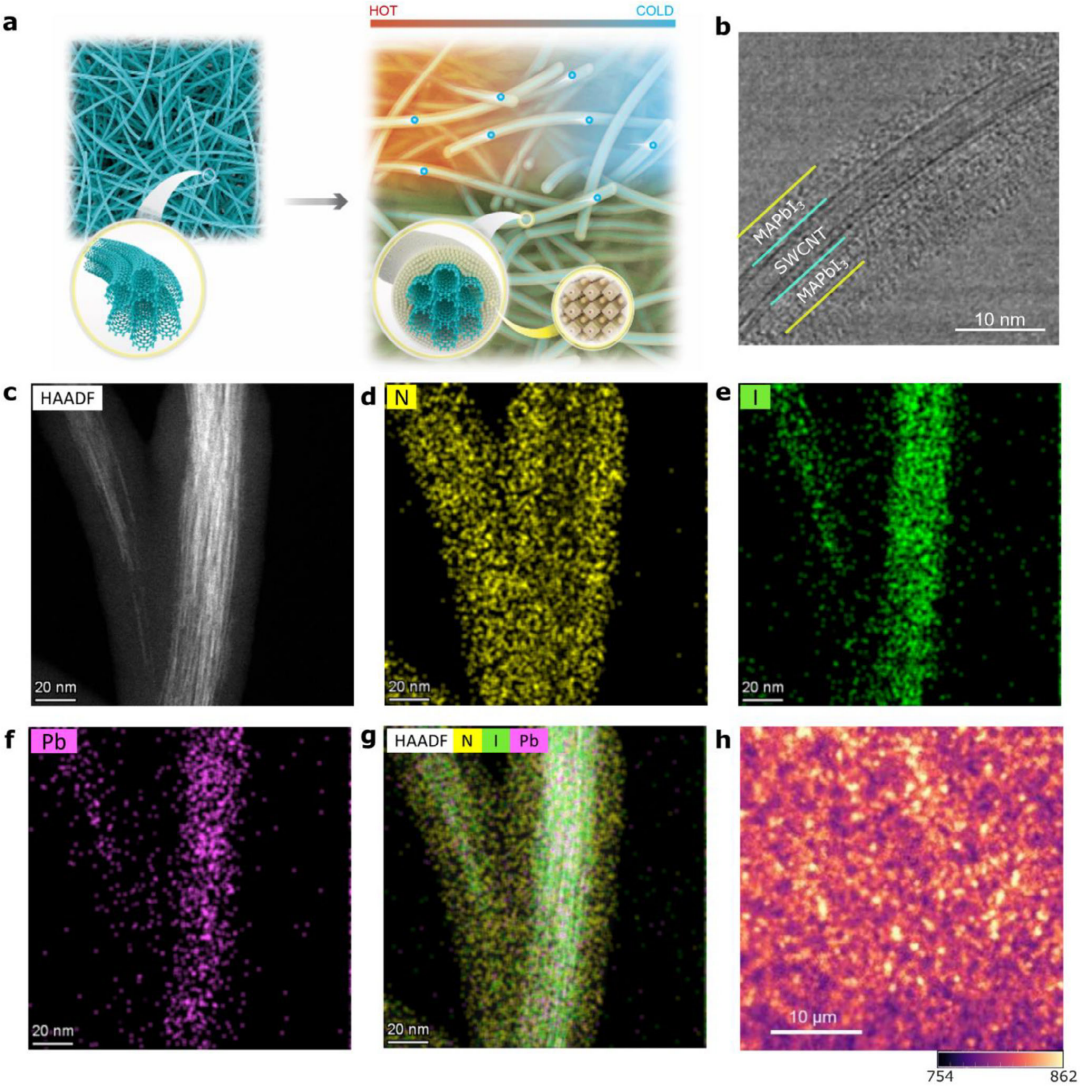
Concept and TEM imaging. a) Schematic illustration of the structure of MAPbI_3_‐coated SWCNT hybrid films under a thermal gradient. b) High‐resolution TEM and c) high‐angle annular dark‐field scanning TEM image (HAADF‐STEM) of SWCNT/MAPbI_3_ show coating of SWCNTs by MAPbI_3_. EDS elemental maps of d) nitrogen (N), e) iodine (I), f) lead (Pb) of SWCNT/MAPbI_3_ exhibit homogeneous coating of MAPbI_3_ on SWCNT. g) Superimposed HAADF‐STEM image and EDS elemental maps of SWCNT/MAPbI_3_. h) Hyperspectral PL image of SWCNT/MAPbI_3_ with emission from MAPbI_3_ showing the coverage of perovskite.

SWCNT/MAPbI_3_ heterostructure films were fabricated using a two‐step approach (Figure S1, Supporting Information), where the SWCNT layer was first deposited via drop casting and the SWCNTs were then coated with MAPbI_3_ by spin coating. The drop casting technique results in randomly oriented and entangled individual and bundled SWCNTs. We hypothesized that the ultralow MAPbI_3_ thermal conductivity should generate a good thermal gradient in the hybrid films, with the long transmission paths in 1D SWCNTs aiding in carrier transport (**Figure** **1**a). Single phase MAPbI_3_ is formed on SWCNTs with broadened X‐ray diffraction (XRD) peaks and reduced crystallinity compared to pristine MAPbI_3_ (Figure S2, Supporting Information). Temperature‐dependent in situ XRD demonstrates a slight decrease in the structural phase transition (tetragonal to cubic) temperature of MAPbI_3_ when coated on SWCNTs (Figure S3, Supporting Information). Top‐view scanning electron microscopy (SEM) shows randomly oriented pristine SWCNT bundles. Addition of the perovskite precursor leads to coating of the SWCNTs with the MAPbI_3_ layer (Figure S4, Supporting Information).

Detailed analysis of the hybrid films by high‐resolution transmission electron microscopy (HRTEM) illustrates the uniform MAPbI_3_ coating of SWCNT bundles. HRTEM image of SWCNT/MAPbI_3_ (Figure 1b) shows a clear outer layer of MAPbI_3_ on the exterior of SWCNT bundles (neat SWCNTs shown in Figure S5, Supporting Information) and two distinct lattice fringes corresponding to each material (Figure S6, Supporting Information). 2D elemental mapping, acquired in high‐angle annular dark‐field scanning TEM (HAADF‐STEM) mode combined with energy‐dispersive X‐ray spectroscopy (EDS) mapping, shows MAPbI_3_ evenly covering the SWCNT surface with uniform spatial distribution of the elements (Figure 1c–g). The coverage of MAPbI_3_ coating on SWCNTs is confirmed by hyperspectral photoluminescence mapping which shows emission around 780 nm from MAPbI_3_ across the film surface (Figure 1h).

### 2.2 Effect of Perovskite on Electrical and Thermal Transport in SWCNTs

**Figure 2:**
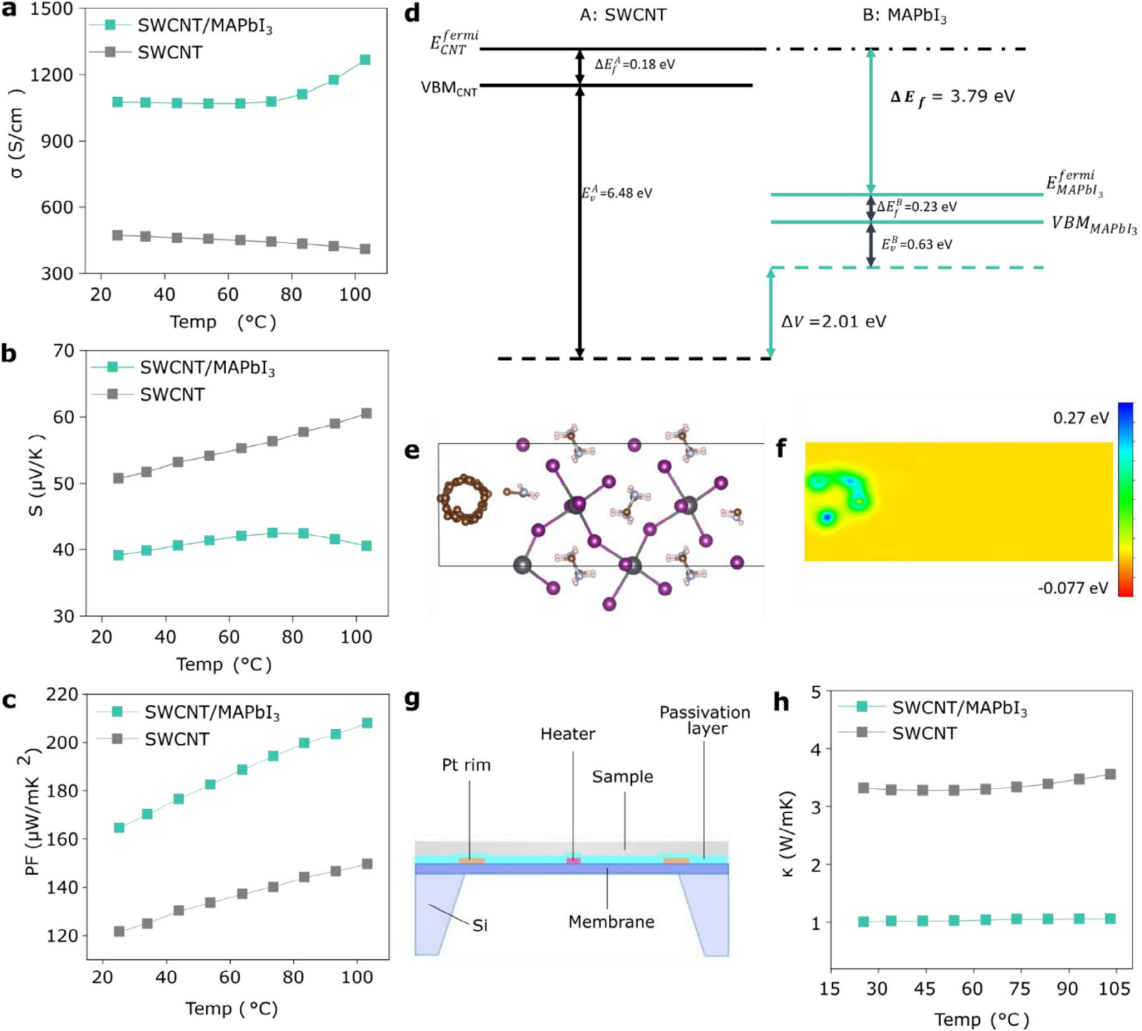
Electrical and thermoelectric characterization. Temperature‐dependent a) electrical conductivity, b) Seebeck coefficient, c) power factor of SWCNT and SWCNT/MAPbI_3_ films. d) Density‐functional‐theory (DFT)‐calculated band alignment at the SWCNT/MAPbI_3_ heterojunction. Energy levels for SWCNT are on the left, with MAPbI_3_ levels on the right. $E_{v(f)}^{A(B)}$ represents the position of the valence band (fermi level) in the bulk with respect to the average electrostatic potential in material *A*(*B*), respectively, and Δ*V* represents the difference between macroscopically averaged electrostatic potential between the two materials (SWCNT and MAPbI_3_) forming the heterojunction. The lower fermi level of MAPbI_3_ (Δ*E*_f_ = 3.79 eV) causes electrons to flow and effectively p‐dope the nanotube. e) Heterojunction between MAPbI_3_ and SWCNT used for f) Bader charge analysis. The charge density difference was calculated as, Δρ = ρ_*t*_ − ρ_MAPbI3_ − ρ_SWCNT_ where ρ denotes charge density obtained through DFT calculation and the subscripts *t*, MAPbI_3_ and SWCNT stand for the combined heterojunction, the structure with only MAPbI_3_, and the structure only with SWCNT, respectively. g) Schematic cross‐sectional view of in‐plane 3ω κ measurement chip. h) In‐plane κ of pristine SWCNT film and SWCNT/MAPbI_3_ film exhibiting significant drop in κ after MAPbI_3_ coating.

To quantify the effect of perovskite coating on the electrical properties of SWCNTs, temperature‐dependent electrical conductivities (*σ*) and Seebeck coefficients (*S*) were measured. The dependence of *S* on the energy distribution of carriers with respect to Fermi level (*E*_f_) can offer insights on carrier type and charge transport mechanisms.^[^ ^28^, ^29^ ^]^ Temperature‐dependent electrical transport properties of pristine SWCNT and SWCNT/MAPbI_3_ films are shown in **Figure** **2**a,b. The room‐temperature *σ* of the SWCNT film increased more than twofold from 472 to 1075 S cm^−1^ after MAPbI_3_ coating, while the *S* decreased from 50 to 39 µV K^−1^, consistent with the expected inverse‐correlation between *σ* and *S*. The positive sign of *S* for both SWCNT and SWCNT/MAPbI_3_ indicates that the majority carriers are holes. The slight decrease in the *σ* for pristine SWCNTs with increasing temperature can be attributed to the desorption of water/oxygen, leading to decrease in hole density.^[^ ^30^ ^]^

The power factor (PF) *σS* ^2^, is a benchmark thermoelectric performance since it correlates with the maximum potential power that can be delivered by a thermoelectric material or device. The highest PF at 103 °C for the SWCNT/MAPbI_3_ film reaches 207 µW m^−1^ K^−2^, an ≈40% improvement relative to the 149 µW m^−1^ K^−2^ PF for the pristine SWCNT film (Figure 2c). There was no performance degradation of the SWCNT/MAPbI_3_ film, even after storage of over 1000 h at ambient conditions (60% RH, 24 °C), underscoring their excellent stability (Figure S7, Supporting Information). Thermogravimetric analysis (TGA) of SWCNT/MAPbI_3_ performed over the same temperature range as electrical measurements shows no degradation, confirming their thermal stability (Figure S8, Supporting Information). The enhancement of *σ* in SWCNT/MAPbI_3_ films was confirmed to be primarily due to the MAPbI_3_ coating (Figure S9 and Note S1, Supporting Information).

The decrease in *S* after coating SWCNTs with MAPbI_3_ agrees with a mechanism in which *E*_f_ moves closer to the valence band and is consistent with p‐type doping of the SWCNTs. Raman‐active SWCNT vibrational modes are sensitive to the SWCNT structure and surroundings. The SWCNT G band is particularly sensitive to charge transfer. Specifically, the G band of p‐type (n‐type) doped SWCNTs is expected to shift to higher (lower) energy, due to mode stiffening (softening).^[^ ^20^, ^31^, ^32^ ^]^ The G band for our SWCNT/MAPbI_3_ film shifts to higher energy compared to pristine SWCNT film (Figure S10, Supporting Information), confirming the p‐type doping. This indicates that ground‐state charge transfer occurs in the SWCNT/MAPbI_3_ heterostructures, leading to excess (majority) holes in SWCNTs. These excess holes should enhance *σ* and decrease *S* (due to p doping), which is consistent with the electrical transport measurements. This behavior of MAPbI_3_ indicates that it acts as a strong electron‐withdrawing group in the heterostructure. The *σ* of SWCNT/MAPbI_3_ films increased with increasing perovskite concentration (Figure S11, Supporting Information), confirming the enhanced hole conductivity is mainly due to increased hole density as a result of p doping.^[^ ^33^ ^]^ Figure 2d shows the band alignment at the SWCNT/MAPbI_3_ heterojunction using density functional theory (DFT) calculations. The MAPbI_3_ *E*_f_ is lower than that of the SWCNT, suggesting a driving force for electron transfer from SWCNT to MAPbI_3_. Bader charge analysis of the SWCNT–MAPbI_3_ heterojunction (Figure 2e) shows excess positive charge on the SWCNT (positive green regions in Figure 2f), confirming the experimentally observed SWCNT p‐type doping.

Thermal conductivity (κ) is known to be high for individual SWCNTs, primarily due to a large lattice contribution. However, randomly oriented networks of SWCNTs exhibit much lower values than individual tubes, as a result of intertube thermal junction resistance.^[^ ^34^ ^]^ Despite achieving impressive *σ* and *S* for SWCNT films, their high in‐plane κ can limit *ZT*.^[^ ^35^, ^36^ ^]^ Forming SWCNT heterostructures has enabled tremendous reduction in κ while maintaining good values for *σ* and *S*, beneficial for thermoelectrics.^[^ ^9^ ^]^ To characterize the effect of conformal perovskite coatings on SWCNT thermal transport, in‐plane κ was measured using a chip‐based 3ω Völklein method (Figure 2g). Coating MAPbI_3_ on SWCNT films significantly reduces κ from 3.3 W m^−1^ K^−1^ for pristine SWCNT films to 1 W m^−1^ K^−1^ for SWCNT/MAPbI_3_ films (Figure 2h). Thus, adding MAPbI_3_ to SWCNTs increases hole density while simultaneously reducing κ, decoupling *σ* and κ. The electronic contribution to the thermal conductivity (κ_e_), estimated from the Wiedemann–Franz law, increases regardless of the Lorenz number used, which was varied between the degenerate limit (Sommerfeld value) to the nondegenerate limit.^[^ ^37^, ^38^ ^]^ This result, combined with the decrease in the total κ, indicates that the lattice thermal conductivity contribution (κ_l_) must decrease in this heterostructure (Figure S12, Supporting Information). Ion migration can impact the measured *σ* and *S*, as can be observed via hysteresis effects in solar cells^[^ ^39^ ^]^ and via giant *S* in ionic thermoelectric devices.^[^ ^40^ ^]^ A recent study demonstrated giant ionic *S* in perovskites over a narrow temperature range around 278 K, but this transient effect required first freezing the sample at 268 K and ramping past 278 K.^[^ ^41^ ^]^ Giant ionic *S* is a well‐known transient effect, and it is especially crucial to allow sufficient equilibration time during *S* measurements, since these transients quickly give way to the steady‐state electronic *S*. Importantly, our thermopower equilibration time is 40 min at each temperature, which avoids any contribution of ionic *S*. As is known from the literature on hysteresis in perovskite solar cells, ion accumulation at interfaces can potentially affect electrical transport via the modulation of thermodynamic barriers and doping of adjacent transport materials. Since the dominant transport material in our nanostructured devices is the CNT phase, it is feasible that some degree of ion accumulation at the CNT/perovskite interface impacts the measured *σ* within the CNT phase. In our recent demonstration of a CNT/perovskite nanocrystal optical synapse, we conjectured that ion accumulation could potentially dope the CNT conducting channel to improve its p‐type conductivity.^[^ ^26^ ^]^ Less is known about the impact of such ion accumulation on κ in perovskite heterostructure interfaces and composites. The above discussion leaves open the possibility that ion migration may impact the *σ* and κ in our composites to some degree, but importantly we utilize long equilibration times that can rule out any ionic contribution to an enhanced *S*.

**Table** **1** summarizes the thermoelectric parameters of SWCNT and SWCNT/MAPbI_3_ films for the temperature corresponding to the highest *ZT*. The calculated in‐plane *ZT* for SWCNT/MAPbI_3_ film reaches 0.07 at 103 °C which is 7 times higher than the pristine SWCNT film (Figure S13, Supporting Information). Additionally, room‐temperature *ZT* of the heterostructure film is ≈10^7^ times higher than pristine MAPbI_3_.^[^ ^42^ ^]^ While the *ZT* value in the present work is modest, the decoupled *σ* and κ, and halide perovskite as a dopant provides a new strategy for perovskite and SWCNT thermoelectrics. Other potential material systems to explore for such decoupling effect might include CNT/quantum dots and perovskite/quantum dots with tunable transport regimes.^[^ ^43^ ^]^ To demonstrate the potential of the present system in flexible electronics, we deposited films on polyethylene terephthalate substrates. No performance degradation was observed in the flexible device after 1000 bending cycles (Figure S14, Supporting Information).

**Table 1:** Thermoelectric parameters of SWCNT and SWCNT/MAPbI3 films corresponding to the highest *ZT*.

Sample	Temperature [°C]	*σ* [S cm^−1^]	*S* [µVK^−1^]	κ [W m^−1^ K^−1^]	PF [µW m^−1^ K^−2^]	*ZT*
SWCNT	103	408.8	60.5	3.56	149.6	0.01
SWCNT/MAPbI_3_	103	1266.2	40.5	1.06	207.8	0.07

To verify the doping effect of MAPbI_3_ on SWCNTs observed in the present work is not unique to a single type of SWCNT, we tested a total of 6 variants of SWCNTs with different diameters and synthesis methods. The doping effect was observed for all SWCNTs types though the magnitude was different, which can be attributed to their diverse structural and electronic properties (Figure S15 and Table S1, Supporting Information). It should be noted that the dispersion behavior and morphology of SWCNTs also play a role in determining the overall electrical performance. Within the scope of the present work, we found that raw (unpurified), longer SWCNTs with an average diameter of 1.3–1.6 nm show the highest doping effect (Figure S16 and Table S2, Supporting Information). These variations among the different SWCNT source materials and diameters warrant further exploration in future studies.

### 2.3 Origin of the Suppressed Thermal Transport in SWCNT–Perovskite Heterostructures

**Figure 3:**
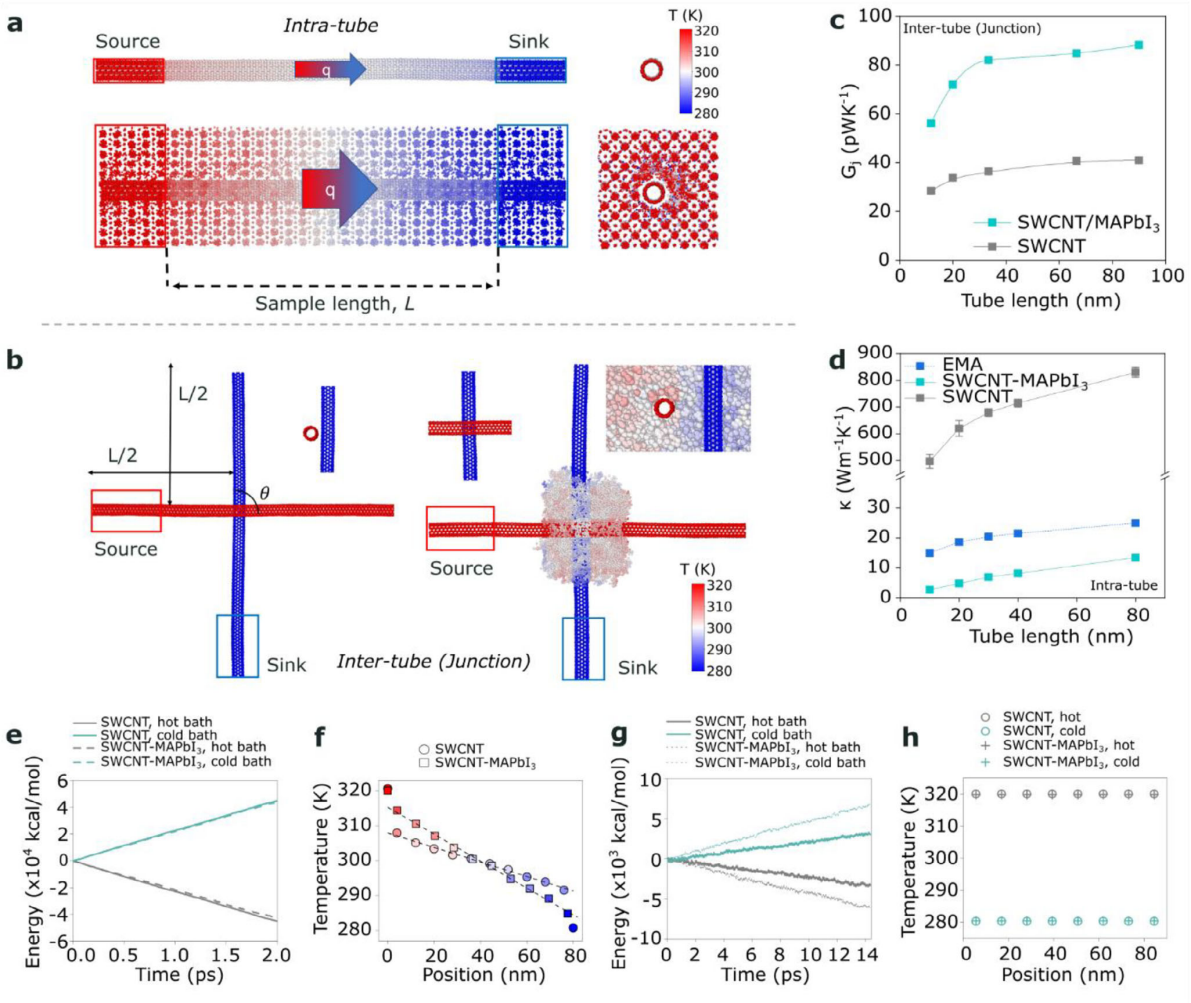
MD simulation setup of a) uncoated SWCNT and SWCNT–MAPbI_3_ and b) uncoated and SWCNT/MAPbI_3_ junction. The color of atoms represents their temperature, with the color bar shown on the right side. The inset of (b) shows the temperature of SWCNT junction underneath the MAPbI_3_ coating, as well as a cross‐section view of the coated junction. c) MD‐calculated thermal conductance of uncoated SWCNT junction and SWCNT/MAPbI_3_ junction and d) MD‐calculated κ of uncoated SWCNT and SWCNT–MAPbI_3_ along with the EMA result. Some of the uncertainty bars are too small to be seen in the figure. e) The energy flowing in and out of the thermal baths with respect to time, for an uncoated and MAPbI_3_‐coated SWCNT. f) The steady‐state temperature distribution along an uncoated and MAPbI_3_‐coated SWCNT. g) The energy flowing in and out of the thermal baths with respect to time, for an uncoated and MAPbI_3_‐coated SWCNT junction. h) The steady‐state temperature distribution of the hot and cold SWCNT in an uncoated and MAPbI_3_‐coated SWCNT junction.

The drop in κ upon doping‐induced increase in carrier density has been previously reported while employing organometallic and organic acid dopants.^[^ ^10^, ^34^, ^44^ ^]^ Despite empirical observations of this technologically relevant effect that can help to decouple *σ* and κ, the fundamental understanding of this behavior is still in the early stages. As such, we employed advanced theoretical tools to model this behavior and provide a much deeper understanding of the interfacial phenomena that can reduce κ in SWCNT‐based heterostructures without negatively impacting *σ*. We first performed nonequilibrium molecular dynamics (NEMD) simulations (see Note S2 and Figure S17 in the Supporting Information for calculation flowchart) and reproduced the reduced κ of the SWCNT/MAPbI_3_ films observed experimentally (details discussed later). We next examined the effect of MAPbI_3_ coating on both a single SWCNT (intratube, **Figure** **3**a) and at a SWCNT/SWCNT junction (intertube, Figure 3b) to understand the origin of this reduced κ. The temperature profile along the CNT in Figure 3a is as expected in a NEMD simulation, with higher temperatures at the hot bath. For the uncoated CNT junction in Figure 3b, the temperature in a single CNT is uniform, at the temperature of the heat bath due to its high thermal conductivity consistent with previous studies.^[^ ^45^ ^]^ For the coated CNT junction, the temperature along a single CNT is identical to the uncoated case. The temperature in the perovskite is influenced by the CNTs’ temperature, with a larger portion of the higher temperature regions around the hot CNT. Due to the limited number of atoms available for local temperature averaging, the temperature fluctuation in the perovskite is large.

**Figure 4:**
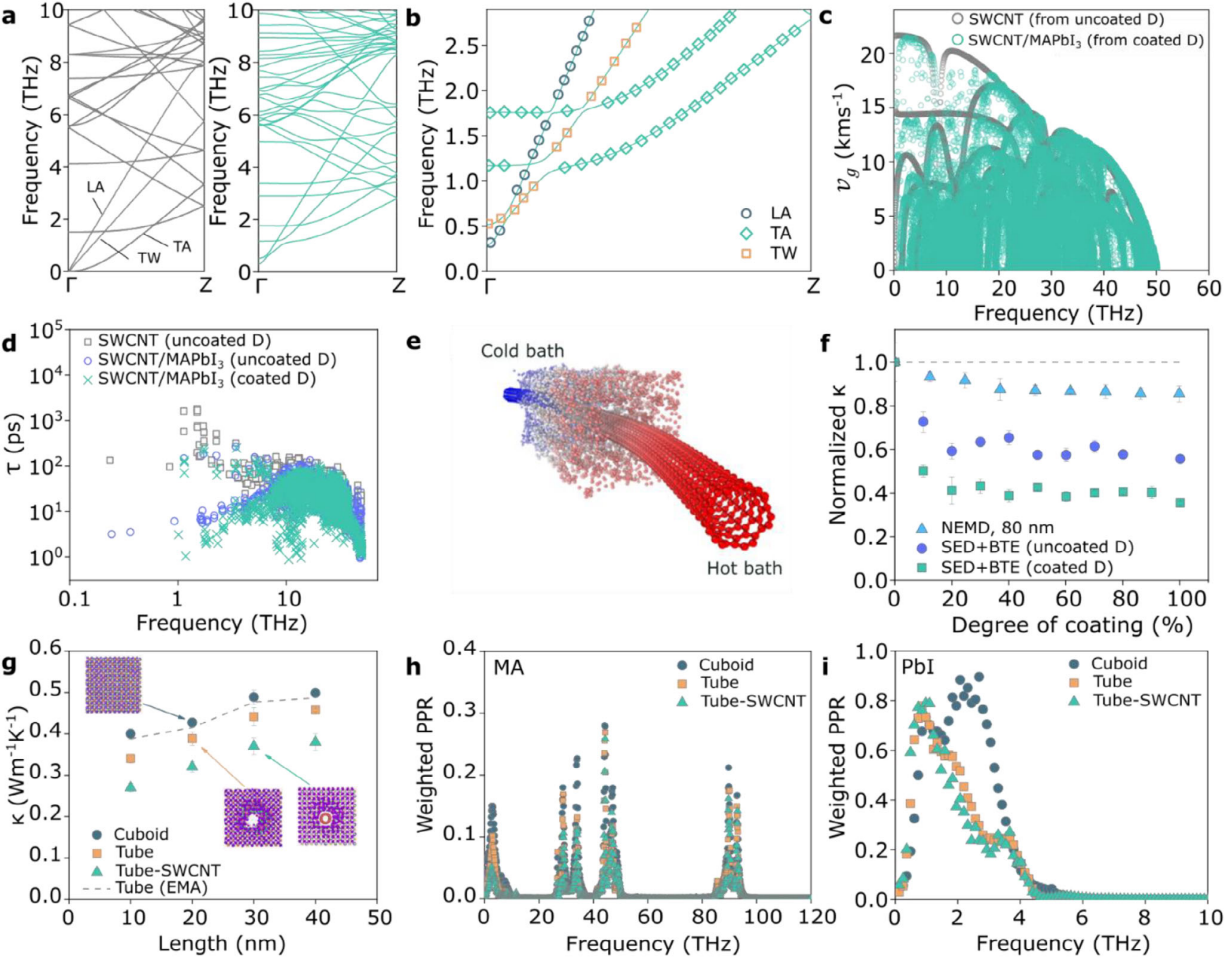
Phonon characteristics. a) Phonon dispersion of a SWCNT that is uncoated (left) and MAPbI_3_‐coated (right). b) Zoom‐in view of the low‐frequency SWCNT phonon modes in the MAPbI_3_‐coated SWCNT, showing the lifting of all acoustic modes near the zone‐center. LA, TW, and TA refer to longitudinal acoustic modes, twisting modes, transverse acoustic modes, respectively. c) Phonon group velocity of uncoated SWCNT and MAPbI_3_‐coated SWCNT. d) Phonon relaxation time of uncoated SWCNT and coated SWCNT calculated using the uncoated D and coated D. e) Schematic of a partially coated MAPbI_3_–SWCNT. f) Normalized κ of the partially coated MAPbI_3_–SWCNT from NEMD and SED + BTE. g) κ of the MAPbI_3_ in the Cuboid, Tube, and Tube–SWCNT cases. The κ of the Tube from an effective medium approximation model κ_tube,EMA_ is shown as dashed line for comparison. h) Density‐of‐states‐weighted phonon participation ratio of the MA molecules. i) Density‐of‐states‐weighted phonon participation ratio of Pb and I atoms.

The intertube thermal conductance values (*G*_j_) for both the uncoated and SWCNT/MAPbI_3_ junctions (Figure 3c) increase with increasing tube length before plateauing. The uncoated *G*_j_ trend agrees with a prior report, showing that long wavelength phonons can contribute significantly to the thermal transport across the junction.^[^ ^45^ ^]^ Coating the SWCNT junction with MAPbI_3_ increases the junction thermal conductance by more than 100%, as the phonons can couple better across the SWCNTs through MAPbI_3_ than vacuum. Although the intratube κ of the uncoated SWCNT(κ_SWCNT_) and the SWCNT–MAPbI_3_ heterostructure (κ_SWCNT − MAPbI3_) increases with length,^[^ ^46^ ^]^ the κ_SWCNT − MAPbI3_ value is substantially smaller, by nearly two orders of magnitude (Figure 3d). For the tube, although the MAPbI_3_ coating can serve as an additional heat conducting channel, the energy transfer rate in and out of the heat reservoirs decreased slightly after coating (Figure 3e). This observation suggests that the interaction between SWCNT and MAPbI_3_ may impede each other's heat transfer, which is confirmed by the larger temperature drop after coating, implying a lower κ in the SWCNT–MAPbI_3_ (Figure 3f). For the junction, the energy transfer rate in and out of the heat reservoirs noticeably increases after coating, due to an ≈100% increase in *G*_j_ (Figure 3g). The temperature distribution is uniform in each SWCNT regardless of any coating as heat prefers to flow through the SWCNT than to cross the junction due to the high SWCNT thermal conductivity (Figure 3h). Also shown in Figure 3d is the κ_SWCNT − MAPbI3_ calculated using an effective medium approximation (EMA) model, where κ_EMA_ =  κ_SWCNT_ × *A*_SWCNT_ + κ_MAPbI3_ × *A*_MAPbI3_ and *A* is the ratio of the cross‐sectional area of the respective constituent. All values for calculating the κ_EMA_ are from MD simulations, with the κ_MAPbI3_ shown as “Cuboid” in **Figure** **4**g. The fact that the MD‐calculated κ_SWCNT − MAPbI3_ is lower than κ_EMA_ suggests that additional scattering of heat carriers occurs which can come from the strong phonon boundary scattering at the SWCNT–MAPbI_3_ interface.^[^ ^47^, ^48^, ^49^ ^]^

To compare with the experimental film κ, we estimated the κ of a film consisting of pure SWCNTs and SWCNT/MAPbI_3_ (κ_film_), by combining our simulated values and the phenomenological model.^[^ ^50^ ^]^ This model includes the effect of CNT bundles and their random orientations and requires the intratube κ and intertube *G*_j_ of SWCNT from MD as inputs (see Note S2 and Figure S18 in the Supporting Information). The order of magnitude and trend of κ_film_ obtained from this model are similar to those from the experiment (Figure S19, Supporting Information). This agreement suggests that significant decrease in the intratube κ overwhelms the increase in *G*_j_ of the SWCNT/MAPbI_3_ junction, resulting in overall lower κ of the SWCNT/MAPbI_3_ films. Although CNTs often exist as bundles in experiments, we found that the intrabundle κ and interbundle *G*_j_ of the CNT bundles exhibit the same trends as those of single CNT tubes (Figures S20 and S21, Supporting Information). Therefore, we use the single tube model in the rest of this paper to extract the pertinent transport physics.

With the understanding that the very low κ of SWCNT/MAPbI_3_ films arises predominantly from a reduction in the intratube κ, and given the failure of EMA to capture this intratube conductance, we performed further MD‐based calculations (Note S3, Supporting Information) of the phonon behaviors. The MAPbI_3_ coating has a minor overall impact on the SWCNT phonon density of states across the whole vibrational spectrum. However, the MAPbI_3_ coating damps out the frequency peaks below 4 THz due to interfacial scattering (Figure S22, Supporting Information). The SWCNT phonon dispersion in the coated case becomes denser and shows features of mode hybridization from the avoided‐crossings,^[^ ^51^ ^]^ and the lifting of zone‐center acoustic phonons (Figure 4a,b). Theoretical phonon studies on SWCNTs in the literature often ignore the changes to the phonon dispersion of the SWCNT in the presence of external physical constraints.^[^ ^52^, ^53^ ^]^ However, accounting for such changes is critical to accurately describe thermal transport in analogous systems, as recently shown for supported/encased graphene.^[^ ^54^, ^55^, ^56^, ^57^, ^58^, ^59^ ^]^ Here, we compared the phonon group velocity (*v*_g_) and relaxation time (τ) obtained using the SWCNT dispersion of the uncoated (uncoated D) and coated (coated D) cases. The τ is obtained using the spectral energy density (SED) method. The differences from using these two dispersions are manifested in a reduction in *v*_g_ and τ (Figure 4c,d). The average *v*_g_ decreases by 20% from 4.79 to 3.80 km s^−1^ while τ drops from 37.6 to 17.8 ps after coating. These reductions originate from the enhanced phonon scattering induced by the densification and avoided‐crossing of the phonon branches.^[^ ^60^ ^]^ Thus, by taking into account the changes in the phonon dispersion, both the phonon group velocity and relaxation time are decreased further, resulting in a lower κ for the SWCNT/MAPbI_3_ (Note S4 and Figures S23–S25, Supporting Information). The accumulated κ in Figure S25 (Supporting Information) shows that the sub‐10 THz phonons account for half of the total κ of SWCNT. These phonons experience significant reduction in phonon relaxation time after coating (Figure 3d), resulting in a large drop in the spectral κ in this range.

From the SEM images, we observed that some SWCNTs might be partially coated, similar to the schematic shown in Figure 4e. Since NEMD results are susceptible to size effects, we supplement our study by calculating κ using the Boltzmann transport equation (BTE) under the relaxation time approximation. Regardless of the calculation approach, the κ of the partially coated SWCNT (Figure 4f) drops with increasing coating degree before ultimately plateauing. Our result suggests that a partial coating of 20% can reduce the κ of a SWCNT up to 40–60% of the uncoated case (Note S5, Figure S26, S27 Supporting Information).

The presence of the SWCNT also reduces the κ of MAPbI_3_. Figure 4g shows κ for MAPbI_3_ model systems as a function of length for three different structures – a cuboid (κ_Cuboid_), a cuboid with a hollow tube (Tube, κ_Tube_), and a cuboid with a SWCNT embedded inside the hollow tube (Tube–SWCNT, κ_Tube − SWCNT_). The κ_Tube_ is lower than the κ_Tube,EMA_, calculated using an EMA model, due to increased phonon scattering in the amorphous region. This region is formed by the reconfiguration of MAPbI_3_ atoms around the hollow tube and seems to decrease the κ contribution from both the organic molecules (MA) and inorganic cage (PbI) equally. Embedding a SWCNT into the tube further reduces κ, suggesting additional amorphization and scattering at the SWCNT surface. Here, the κ contribution from MA decreases more significantly (Figure S28, Supporting Information). To understand these differences, the phonon participation ratio (PPR) was calculated in a MAPbI_3_ region within the Lennard Jones interaction cutoff distance from the SWCNT surface. The κ trend followed the PPR trend, where PPR_Cuboid,MA_ > PPR_Tube,MA_ > PPR_Tube–SWCNT,MA_ for frequencies below 25 THz (Figure 4h), signifying an increase in phonon localization near the hollow tube surface. This localization decreases the κ contribution from MA. On the other hand, a redshift in the phonon frequencies of PPR_Tube,PbI_ and PPR_Tube–SWCNT,PbI_ from PPR_Cuboid,PbI_ (Figure 4i) indicates a softening of PbI phonon modes, which reduces the group velocities and lowers the κ.^[^ ^27^ ^]^ Further reduction of κ for PbI in the Tube–SWCNT structure possibly arises from increased scattering at the MAPbI_3_–SWCNT interface (Note S6 and Figures S28–S34, Supporting Information).

To summarize the thermal transport investigation, interactions between the SWCNT and MAPbI_3_ result in a decrease in their respective thermal conductivity, leading to an overall drop in the κ of the heterostructure. The phonon dispersion of the SWCNT changes after coating, reducing the phonon group velocity and relaxation time to a much lower level. These changes reduce the BTE‐predicted κ up to 60% of the uncoated SWCNT value. Results from NEMD and BTE suggest that a partially MAPbI_3_‐coated SWCNT can experience a similar κ reduction as a fully coated SWCNT. The κ of the MAPbI_3_ is reduced by 1) the amorphization of MAPbI_3_ around the SWCNT that localizes the vibrations of the organic cations and induces the softening of the phonons from the inorganic anions; and 2) the interaction with the SWCNT that further localizes the vibrations of the organic cations.

## 3 Conclusions

Decoupling of electrical and thermal conductivity was demonstrated in SWCNT/MAPbI_3_ heterostructures that exhibit excellent long‐term stability. Our results revealed that coating SWCNTs with hybrid perovskites substantially reduces the thermal conductivity of the SWCNTs while increasing their electrical conductivity, offering a promising route for decoupling and tuning the electrical and thermal transport properties of SWCNT heterostructures. The charge transfer mechanism and doping behavior elucidated here can lead to new device concepts and better fundamental understanding of charge transfer between perovskites and SWCNTs. MD simulations and related calculations reveal a decrease in the κ of an individual SWCNT by up to 40–60% after perovskite coating. Such thermal conductivity reduction originates from a lower phonon group velocity and relaxation time. Moreover, coating only 20% of a SWCNT with MAPbI_3_ produces a similar thermal conductivity decrease as a 100%‐coated SWCNT. The κ of the MAPbI_3_ near the SWCNT/MAPbI_3_ interface is reduced due to phonon softening and localization. Tailoring the SWCNT transport properties and ability to harness ultralow thermal conductivity of perovskites opens new avenues for further exploration of this class of materials for near room‐temperature thermoelectrics and other functional devices.

## Conflict of Interest

The authors declare no conflict of interest.

## Author Contributions

M.A.H. and T.Y. contributed equally to this work. M.A.H., W.‐L.O., and D.B. designed the project. T.Y., Y.Z., and W.‐L.O. performed the MD simulations and wrote the analysis. M.A.H. fabricated the samples, performed all thermoelectric measurements. L.H.H. contributed to sample preparation. C.C. performed TGA measurement. H.C. performed the DFT calculations and wrote the analysis, supervised by E.H.S. A.G. performed the TEM characterization. B.D. performed 2D and temperature‐dependent XRD measurements. J.L.B. performed the absorption measurements and analyzed the SWCNT types. H.L., J.M.L., and E.H.S. contributed to analysis, writing, and resources. M.A.H. and T.Y. wrote the initial draft of the paper and all authors contributed to the editing of the paper.

## Supporting information



Supporting Information

## Data Availability

The main data supporting the findings of this study are available within the Article and its Supplementary Information.
