# Supplementary material for: Nanoscale Decoupling of Carrier–Phonon Transport in Carbon Nanotube–Halide Perovskite Heterostructures
Source: Adv Sci (Weinh). 2025 Sep 3;12(43):e07589. doi: 10.1002/advs.202507589 (PMC12631927; doi:10.1002/advs.202507589)
Supplement: Supplementary file 1 — Supporting Information [file ADVS-12-e07589-s001.docx]

Supporting Information

**Nanoscale Decoupling of Carrier-Phonon Transport in Carbon Nanotube-Halide Perovskite Heterostructures**

*Md Azimul Haque,^†^ Taocheng Yu,^†^ Hitarth Choubisa, Luis Huerta Hernandez, Yuan Zhou, Alessandro Genovese, Bambar Davaasuren, Craig Combe, Hanying Li, Joseph M. Luther, Jeffrey L. Blackburn, Edward H. Sargent, Wee-Liat Ong,* Derya Baran**

M. A. Haque, L. H. Hernandez, C. Combe, D. Baran

Material Science and Engineering Program (MSE), Physical Sciences and Engineering Division (PSE), King Abdullah University of Science and Technology (KAUST), Thuwal 23955-6900, Kingdom of Saudi Arabia

Email: derya.baran@kaust.edu.sa

T. Yu, Y. Zhou, W.-L. Ong

ZJU-UIUC Institute, College of Energy Engineering, Zhejiang University, Haining, Jiaxing, Zhejiang 314400, China

Email: weeong@intl.zju.edu.cn,

H. Choubisa, E. H. Sargent

Department of Electrical and Computer Engineering, University of Toronto, 35 St George Street, Toronto, ON M5S 1A4, Canada

A. Genovese, B. Davaasuren

King Abdullah University of Science and Technology (KAUST), Corelabs, Thuwal 23955-6900, Saudi Arabia

M. A. Haque, J. M. Luther, J. L. Blackburn

National Renewable Energy Laboratory, Golden, CO, 80401 USA

H. Li

MOE Key Laboratory of Macromolecular Synthesis and Functionalization, International Research Center for X Polymers, Department of Polymer Science and Engineering, Zhejiang University, Hangzhou 310027, China

W.-L. Ong

State Key Laboratory of Clean Energy Utilization, Zhejiang University, Hangzhou, Zhejiang, 310027, China

^†^These authors contributed equally

**Experimental Section**

**Materials**

PbI_2_ (99.9985%) was purchased from Alfa Aesar and CH_3_NH_3_I from Greatcell Solar. SWCNT were purchased from Sigma (Type 1 and Type 2), NanoIntegris (Type 3 and Type 4), Timesnano (Type 5) and MEIJO (Type 6). More details on SWCNTs are shown in Table S1. All other materials were purchased from Sigma-Aldrich. All chemicals were used as received. All electrical and thermal characterization data are based on Type 5 (Timesnano) SWCNT unless indicated otherwise.

**Sample preparation**

SWCNT films were prepared by drop casting SWCNT dispersion (1 mg/ml) in chloroform on glass substrates. The SWCNT dispersion was ultra-sonicated for 5 minutes before drop casting. The sample size was 25 mm x 10 mm. After all the solvent evaporated from the drop-casted film at room temperature and the films appeared dry, the films were annealed for 10 minutes in air at 100 °C. To prepare the perovskite precursor solution, MAI, and PbI_2_ with required molar ratios were dissolved in DMF and DMSO (a volume ratio of 9:1) with a total concentration of 1.4 M. To coat MAPbI_3_ on the SWCNT films, the precursor solution was spin coated at 1000 rpm for 10 s and 4000 rpm for 30 s in an N_2_ glove box. Spin coating was started after a waiting time of 2 minutes so that precursor solution diffuses into the SWCNT film. Chlorobenzene was dripped on to the spinning substrate after the first 18 s of the spin coating process. The spin-coated films were annealed at 50 °C for 20 minutes and then at 100 °C for 20 minutes. The thickness of SWCNT/MAPbI_3_ films was ̴ 10 µm measured by profilometer.

**Material characterization**

Top-view SEM images were obtained using FEI Nova Nano. For high-resolution transmission and scanning electron microscopy (HRTEM) investigations, we used an FEI Titan 80–300 Cubed microscope equipped with a high-brilliance field emission gun (300 kV), a Wien type FEI monochromator and a spherical aberration (Cs) corrector for the objective lens allowing final spatial resolution of 0.09 nm. For scanning transmission electron microscopy (STEM) and chemical investigations, we used a second FEI Titan 80–300 Cubed microscope equipped with a high-brilliance field emission gun (300 kV), a Wien type FEI monochromator a spherical aberration (Cs) corrector for the condenser lens - allowing final spatial resolution of 0.09 nm in STEM mode – and the ChemiSTEM system for X-ray energy dispersive spectroscopy (EDS) made of four SDD EDX detectors.). Room temperature and in-situ temperature-dependent X-ray diffraction (XRD) patterns were obtained from the Bruker D8 ADVANCE diffractometer equipped with Anton Paar HTK 1200 oven chamber. 2D XRD measurements were carried out on Bruker D8 Discover system equipped with IμS micro focus Cu-source and multimode Eiger2 R 500K 2D detector. UV-Vis absorption spectra of the films were recorded using Perkin-Lambda spectrometer. Raman spectra were recorded using Witec Apyron confocal Raman Imaging system. TGA was carried out using TA Instruments Discovery. The PL image of the sample was collected using a hyperspectral imaging system (Photon etc. IMA).

**Thermoelectric measurements**

Electrical conductivity and Seebeck measurements were performed on Netzsch SBA 548 Nemesis thermoelectric instrument under He environment. First SWCNT films were measured and then the same films were coated with MAPbI_3_ and re-measured to minimize errors. Shelf-life stability was measured by keeping the sample in dark under ambient conditions (24 °C, 60% relative humidity). In-plane was measured under vacuum using the 3*ω* Völklein method with a Linseis Thin Film Analyzer.^[1]^ Samples were prepared onto prepatterned test chips from Linseis by similar method as in case of glass substrates. The measurement uncertainty for *σ*, *S*, and are ±5%, ±7%, and ±7%, respectively.

**DFT for charge transfer**

The values for band-alignment were obtained using the approach described by Weston et. al.,^[2]^ that uses macroscopic potential to find the band offsets of a heterojunction. We used HSE06 exchange-correlation functional with Spin orbit coupling to capture the effects of the heavy elements present. All the calculations were done using VASP^[3]^ with energy cutoff of 520 eV, force convergence criteria of 0.001 eV/atom and k-points grid of $1\times2\times1$. Processing of the output was done using VASPkit.^[4]^


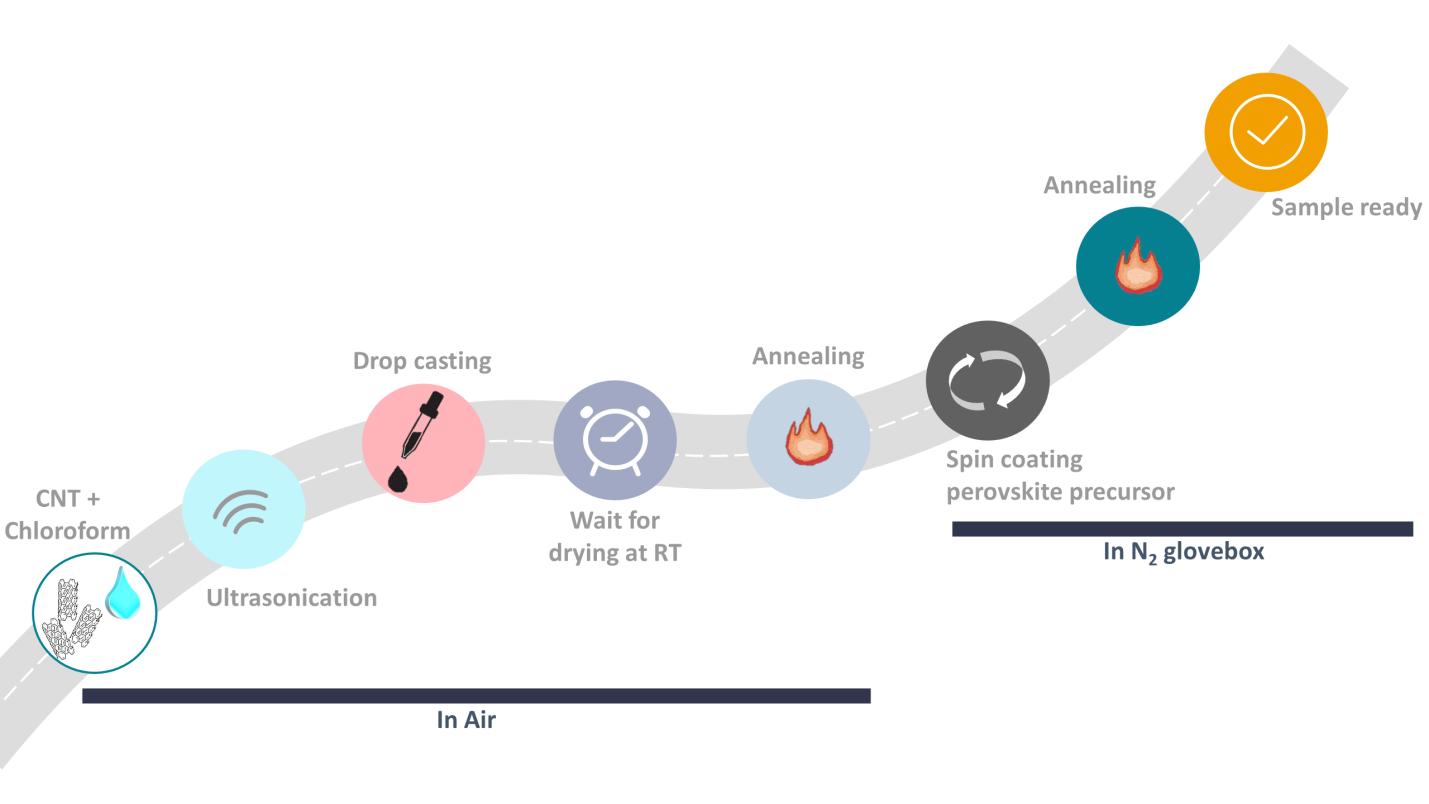


**Fig. S1** Schematic fabrication process of SWCNT/MAPbI_3_ hybrid thermoelectric films.


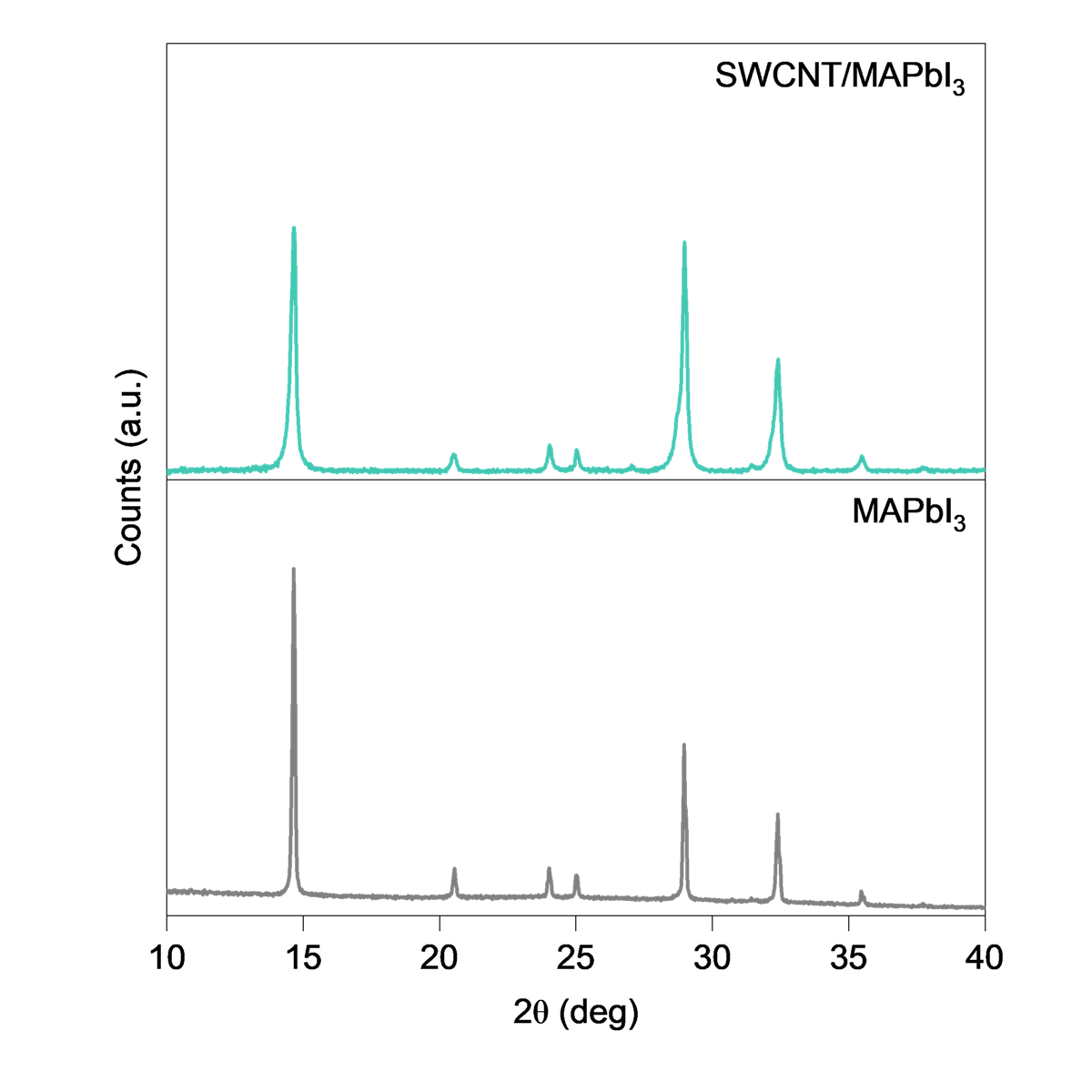


**Fig. S2** XRD patterns for fresh MAPbI_3_ and SWCNT/MAPbI_3_ films.


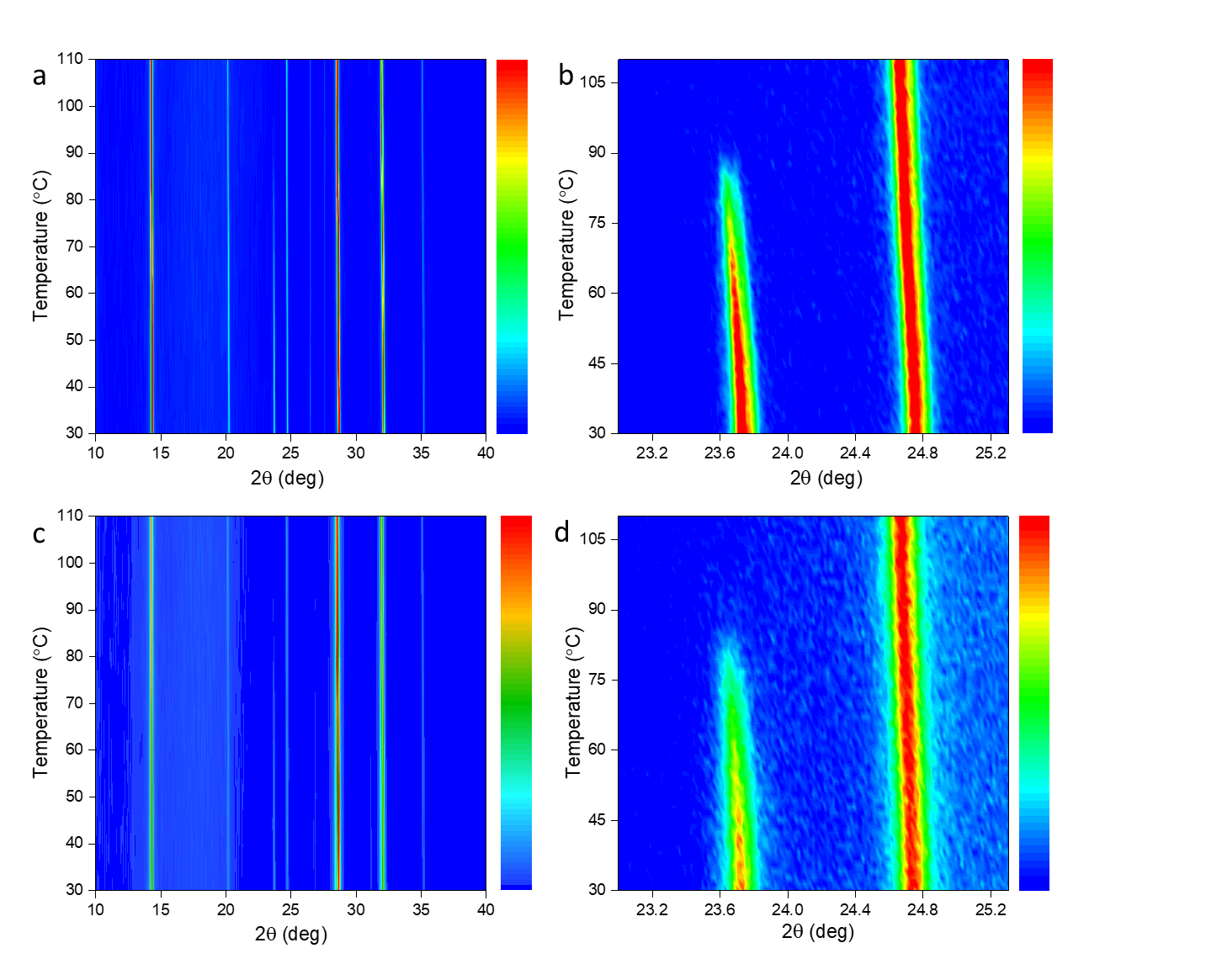


**Fig. S3** In-situ temperature-dependent XRD of **a,b** MAPbI_3_ and **c,d** SWCNT/MAPbI_3_ films. b and d show the phase transition in high resolution.


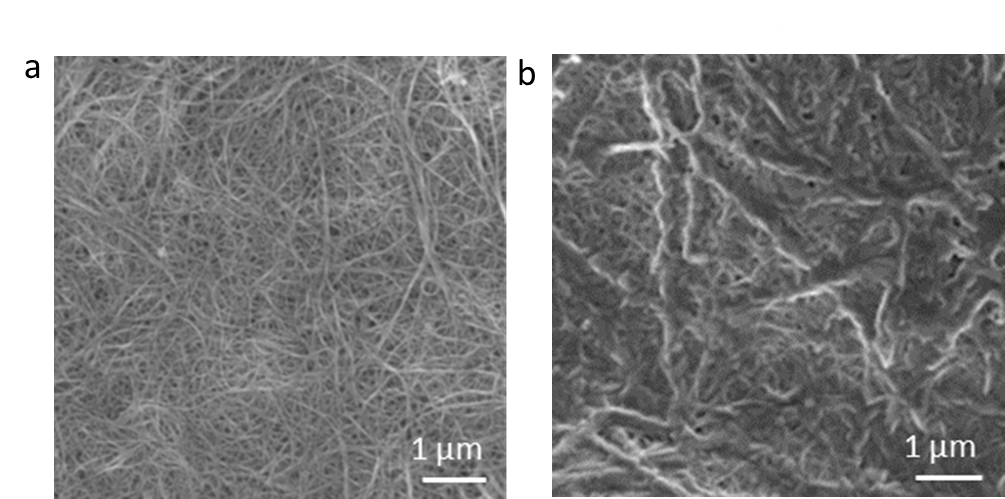


**Fig. S4** SEM images for pristine **a** SWCNT and **b** SWCNT/MAPbI_3_ films.


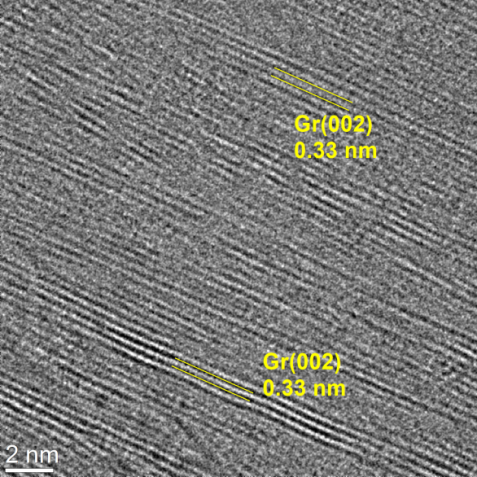


**Fig. S5** HRTEM image of pristine SWCNTs showing they consist of bundles.


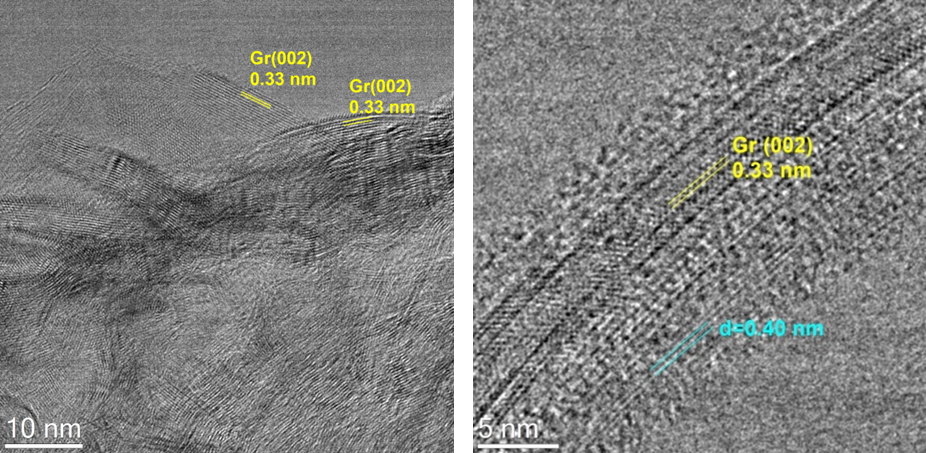


**Fig. S6** HRTEM image of SWCNT/MAPbI_3_. It should be noted that electron beam can induce damage to MAPbI_3_ during imaging.


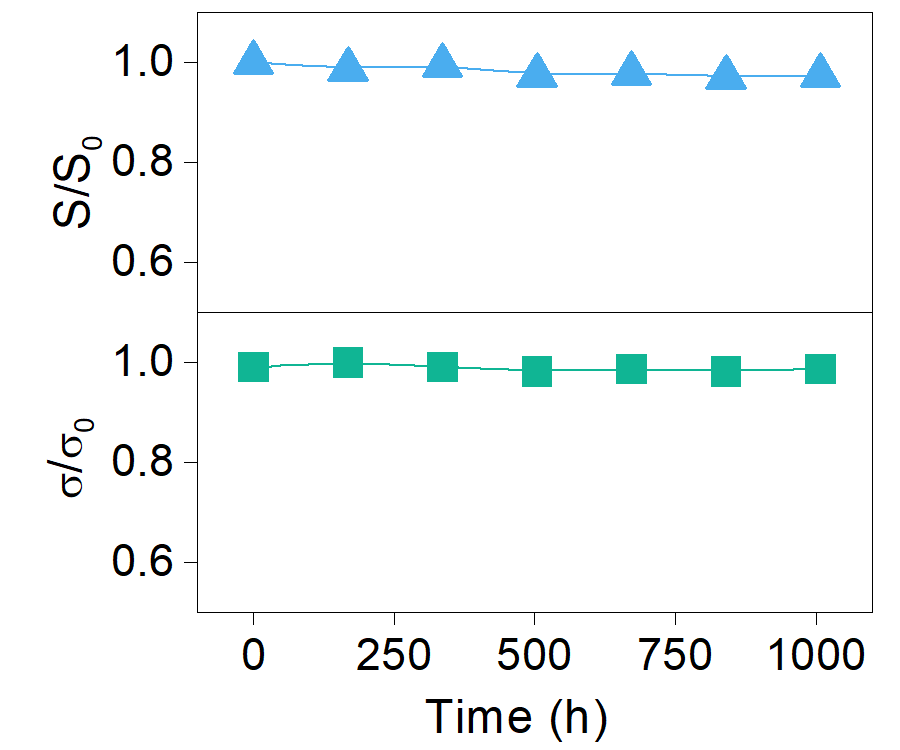


**Fig. S7** Stability of electrical conductivity and Seebeck coefficient after storage in ambient conditions (24 °C, 65% RH).


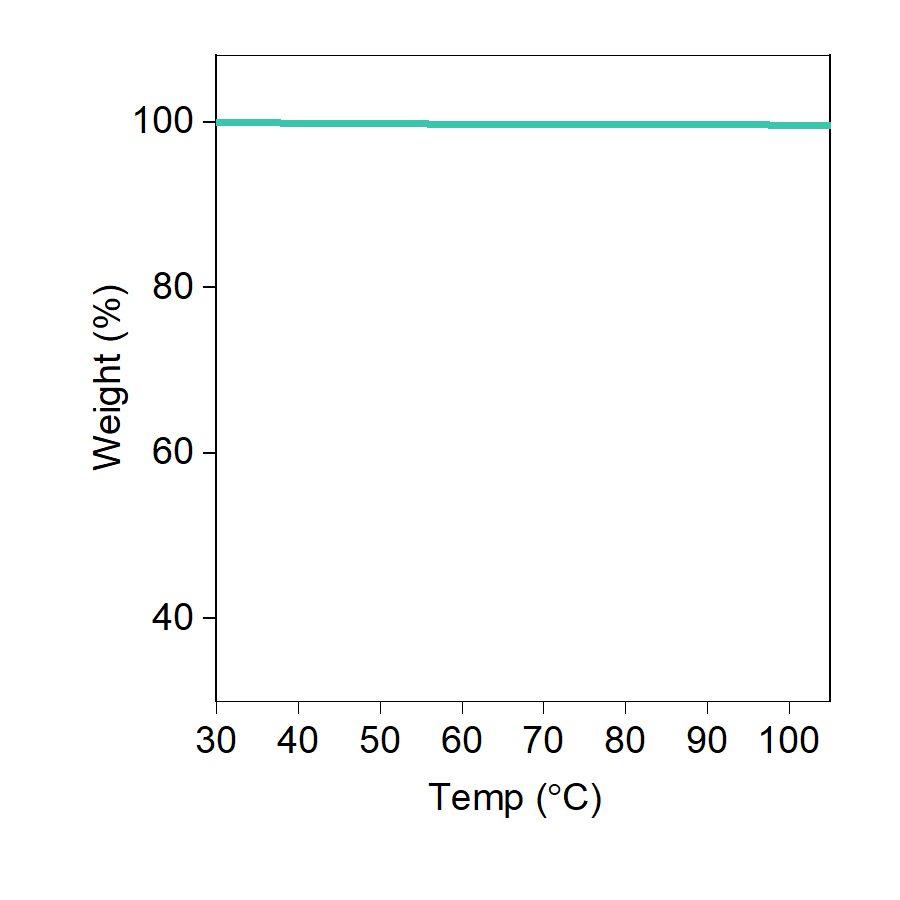


**Fig. S8** TGA curve for SWCNT/MAPbI_3_ showing no degradation.

**Supplementary note 1**

To confirm that the enhancement of *σ* in SWCNT/MAPbI_3_ hybrid films was primarily due to MAPbI_3_ coating, the change in *σ* for the composite films (relative to SWCNTs only) was compared to control samples of SWCNTs treated with individual precursors and solvent (Fig. S9). For solvent treatment of SWCNTs, exactly same solvents and process were used as in case of MAPbI_3_ precursor. Pure DMF/DMSO was spin coated on the SWCNT film and then chlorobenzene was introduced as antisolvent during the process. Interestingly, this solvent treatment results in dedoping of the SWCNT film, relative to some uncontrolled ambient doping, with *σ* lower than the pristine SWCNT film. In contrast, *σ* increased for methylammonium iodide (MAI) and lead iodide (PbI_2_) treated SWCNT films but the *σ* was significantly lower than MAPbI_3_ treated SWCNTs. This comparison strongly suggests that the enhanced performance of SWCNT/MAPbI_3_ hybrid films is due to the interaction between MAPbI_3_ ­and SWCNTs.

**Fig. S9** Change in electrical conductivity of SWCNT coated with only solvent, MAI, PbI_2_ and MAPbI_3_.

**Fig. S10** Raman spectra of pristine SWCNT and SWCNT/MAPbI_3_ films.

**Fig. S11** Electrical conductivity of SWCNT/MAPbI_3_ films with different MAPbI_3_ precursor concentrations.


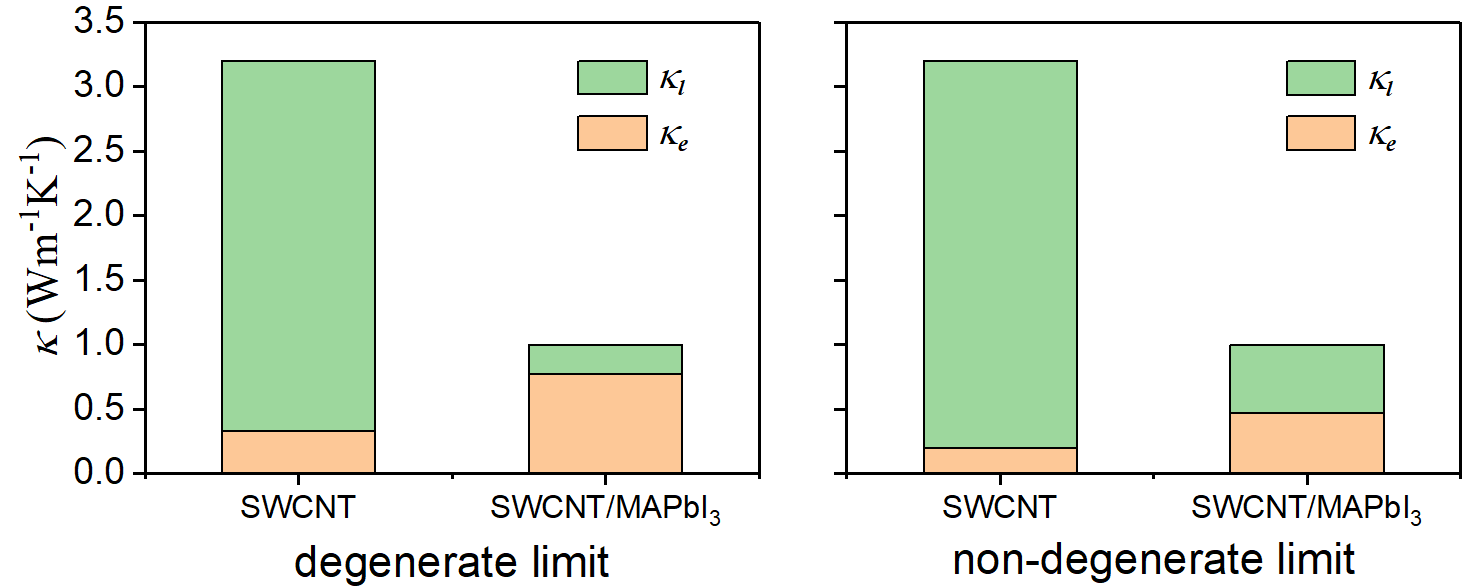


**Fig. S12** Contributions of the electrons (${}_{e}$) and lattice (${}_{l}$) to the total thermal conductivity before and after MAPbI_3_ coating.

**Fig. S13** Figure of merit, ZT for pristine SWCNT and SWCNT/MAPbI_3_ films.


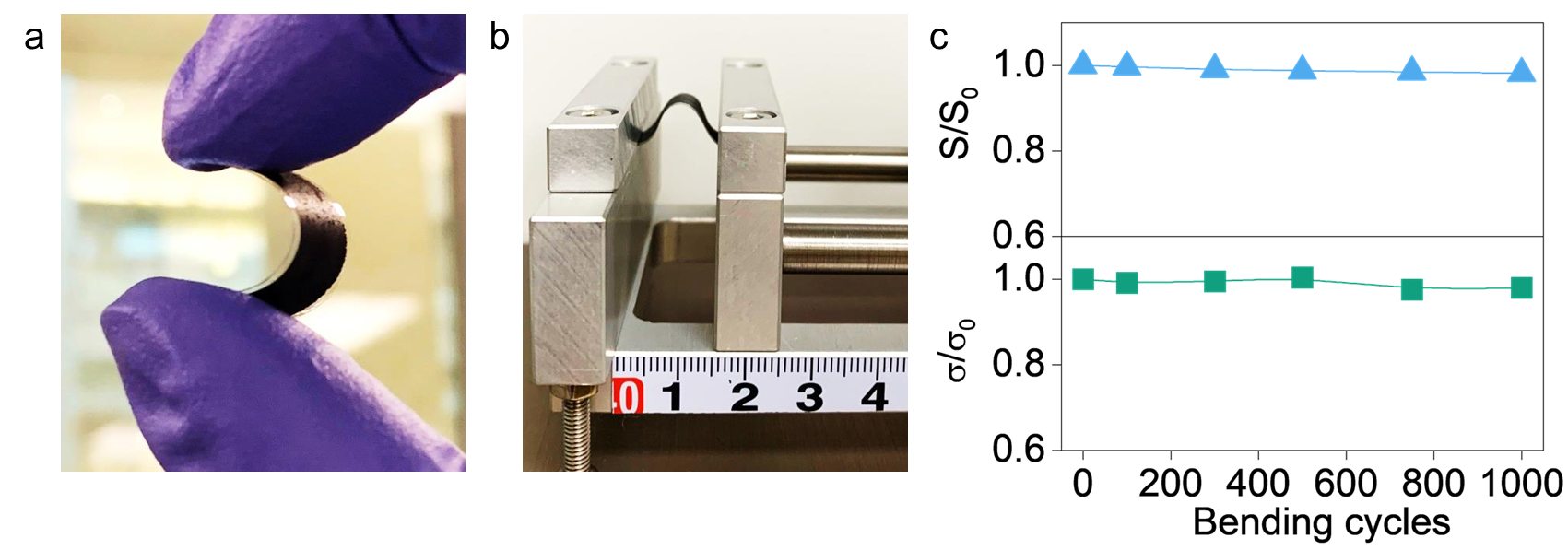


**Fig. S14** Flexible device of SWCNT/MAPbI_3_. Bending radius was 7.5 mm.

**Fig. S15** Electrical conductivity, Seebeck, and power factor of different types of SWCNT and their composite with MAPbI_3_.

**Table S1** Electrical conductivity and Seebeck coefficient of different types of SWCNTs for highest measurement temperature (103 °C).

| Sample | σ (S/cm) | | S (µV/K) | |
| --- | --- | --- | --- | --- |
|  | Pristine | With MAPbI_3_  σ (S/cm) | Pristine | With MAPbI_3_  S (µV/K) |
| Sigma (Type 1) | 107.2 | 212.7 | 33.7 | 32.3 |
| Sigma (Type 2) | 160.3 | 241.2 | 27.5 | 26.1 |
| NI HiPCO (Type 3) | 43.1 | 101.5 | 38.8 | 34.5 |
| NI PT (Type 4) | 20.9 | 1323.2 | 39.7 | 35 |
| Times Nano (Type 5) | 408.8 | 1266.2 | 60.5 | 40.5 |
| MEIJO e-DIPS (Type 6) | 8.8 | 20.6 | 52.1 | 33.5 |


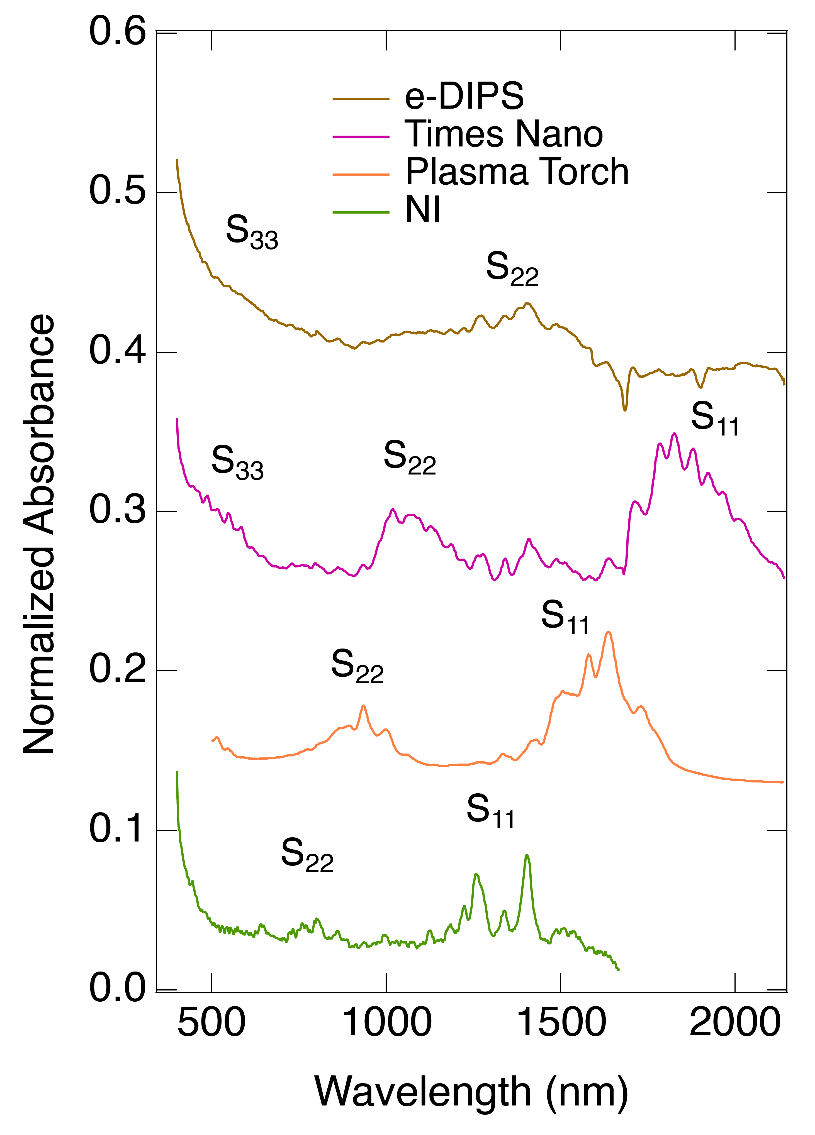


**Fig. S16** Absorption spectra of different types of SWCNTs.

**Table S2** Details of different types of SWCNTs.

| Sample | Growth Method | Avg. Diameter | Purity | Expected Length |
| --- | --- | --- | --- | --- |
| Sigma (Type 1) | Fixed bed CVD  (Cobalt/Moly) | 0.83 nm | purified | Very short (<500 nm) |
| Sigma (Type 2) | Fixed bed CVD  (Cobalt/Moly) | 0.84 nm | purified | Very short (<500 nm) |
| NI HiPCO (Type 3) | Fixed bed CVD  (Fe- CO) | 1.1 nm | purified | Short (< 1 μm) |
| NI Plasma Torch (Type 4) | Plasma Torch | 1.3 nm | Raw | Long (> 2 μm) |
| Times Nano (Type 5) | FCCVD | 1.6 nm | Raw | Long (> 2 μm) |
| MEIJO e-DIPS (Type 6) | FCCVD | 2 nm | Raw | Long (> 2 μm) |

**Supplementary note 2**

**1. The MD simulation**

**i) Thermal conductance of a SWCNT junction and MAPbI_3_-coated SWCNT junction**

For a given junction configuration, two SWCNTs of the same length (*L*) forms a perpendicular junction located at the center of each SWCNT. The atomic interaction within each SWCNT is described using the optimized Tersoff potential^[5]^ while the interaction between the SWCNTs is approximated by a 12-6 Lennard Jones potential with *σ* = 3.4 Å and *ε* = 2.84 meV.^[6]^ To investigate the effect of SWCNT length on the junction thermal conductance, the length of each SWCNT was varied from 10 nm to 90 nm.

For the MAPbI_3_-coated SWCNT junction, a 9 x 6 x 4 unit-cell of tetragonal MAPbI_3_ was used to wrap around the SWCNT junction. Two perpendicular tunnels, each with a cross-sectional area of 2 x 2 unit-cell, were carefully created to maintain charge neutrality in the MAPbI_3_ for inserting the SWCNTs (Fig. 3b). One unit-cell of MAPbI_3_ separated these two SWCNTs. The MYP potential was used to describe the interactions in the MAPbI_3_.^[7]^ This potential has successfully reproduced experimental temperature-dependent thermal conductivity trend and phase changes of MAPbI_3_ in several studies.^[8-10]^ As a first approximation, the interactions between the carbon atoms in the SWCNT and MAPbI_3_ are described by a 12-6 Lennard Jones (LJ) potential, with the associated parameters obtained using the Lorentz-Berthelot mixing rule.^[11]^

The MD simulation was performed in LAMMPS^[12]^ with a time step of 0.5 fs. The periodic boundary condition was adopted in all directions. The system was first equilibrated in NPT at a temperature (*T*) of 300 K and 0 bar for 2 ns using the Nose-Hoover thermostat and further in NVT for 2 ns to ensure that the SWCNTs and MAPbI_3_ are at 300 K. The system was then switched to NVE for 2 ns to ensure system stability. The relaxed cross-section of the junction (inset in Fig. 3b) shows some disorder in the MAPbI_3_ near the SWCNTs. After the equilibration step, a 1 nm segment at each end of the SWCNTs was fixed and the boundaries of the simulation box were lengthened by 10 nm to remove interactions across the periodic boundary. The NEMD method was then used to calculate the junction conductance. The Langevin thermostat was used to maintain a heat source at *T* = 320 K or a heat sink at *T* = 280 K on a 5 nm SWCNT segment adjacent to one of the fixed ends in each of the two SWCNTs. Steady-state was achieved after 5 ns. The steady-state temperature gradient along each SWCNT was calculated by averaging over the next 20 to 30 ns. As the heat (*q*) flows across only at the junction resulting in a temperature drop of *ΔT* across the two SWCNTs, this junction thermal conductance ($G_{j}$) can be calculated as $G_{j}$ = *q* ∕ *ΔT*.

**ii) Thermal conductivity of an uncoated SWCNT and SWCNT-MAPbI_3_ heterostructure**

Using a 6 x 6 unit-cell cross-sectional area tetragonal phase MAPbI_3_, a square tunnel of a 2 x 2 unit-cell was carefully removed to embed a SWCNT (Fig. 3a). The length (*L*) of the composite was varied from 10 nm to 80 nm. Corresponding MAPbI_3_ systems without the tunnels were built for the EMA calculation. The potentials used are identical to those in the simulation of the SWCNT junctions. These systems were relaxed and equilibrated following the procedures in the above section. The relaxed cross-section of the SWCNT-MAPbI_3_ in the Fig. 3a shows some disorder in the MAPbI_3_ near the SWCNTs. A segment of about 1.2 nm at each end was fixed before the NEMD step.

The thermal conductivity of these systems was calculated using the NEMD method with a 5 nm long heat source and sink applied near each ends of the system. These heat source and sink were controlled at *T* = 320 K and *T* = 280 K with a Langevin thermostat (damping constant of 10 fs). Steady-state was reached after 5 ns. The temperature gradient was calculated using eight to ten equally divided regions along the SWCNT-MAPbI_3_ averaged over the next 20 to 30 ns and used in the Fourier Law to calculate the thermal conductivity.

**2. Calculation of** ${}_{\boldsymbol{film}}$

Using the junction thermal conductance and thermal conductivity of a SWCNT from our simulations, we adopted a recently published model to estimate the thermal conductivity of a film of SWCNTs.^[13]^ In that published model, the SWCNT film model has a size of 100 μm × 100 μm, with a transparency 87% that is equivalent to a line density ($D_{L}$) 38 ${\mu m}^{-1}$. The heat conducting paths in the film from the hot to cold baths are composed of overlapping bundles of SWCNTs with junctions connecting them from end to end. They used the theoretical model developed by Pop et al. to calculate the thermal conductivity of SWCNT and equation (S1) to account for the reduction in thermal conductivity for a SWCNT bundle.^[14]^

${}_{b}=\frac{{}_{is}}{Z}$ (S1)

where ${}_{b}$ and ${}_{is}$ are the thermal conductivity of a SWCNT bundle and individual SWCNT, respectively. $Z$ is factor related to the number of SWCNT in a bundle ($n_{b}$) and the temperature (*T*). Using Monte Carlo simulations, they verified the accuracy of equation (S2) to calculate the thermal conductance of a path ($G_{P}$), where $G_{j}$ is the junction thermal conductance, $G_{b}$ is the bundle thermal conductance and $N_{j}$ is the number of junctions per path.

$G_{P}=\left( \left( N_{j}+1 \right)\times\frac{1}{G_{b}}+N_{j}\times\frac{1}{G_{j}} \right)^{-1}$ (S2)

The thermal conductance of a SWCNT film can be calculated from equation (S3)

$G_{f}= \alpha\times D_{L}\times G_{p}$ (S3)

where the factor $\alpha$ is obtained by fitting the model with experimental measurement.^[15]^

We modeled the above SWCNT film with the same geometrical parameters and replaced the appropriate parameters from our MD results. Due to the large computational cost, we calculated the thermal conductivity of the SWCNT-MAPbI_3_ up to a length of 120 nm, which involved more than 180 000 atoms. We carried out Monte Carlo simulation to determine the number of junctions per path. From our MD simulations, the $G_{j}$ of both uncoated and MAPbI_3_-coated SWCNT junction increases before plateauing with increasing SWCNT length. This converged thermal conductance value of the junction was used for subsequent calculation of the SWCNT film as the length of SWCNT in the experiments is much longer (5 μm~30 μm). The cross-sectional area of SWCNT film $A_{f}$ is the product of the film width $L_{f}$ and the film thickness. The above procedure is summarized in Fig. S17.


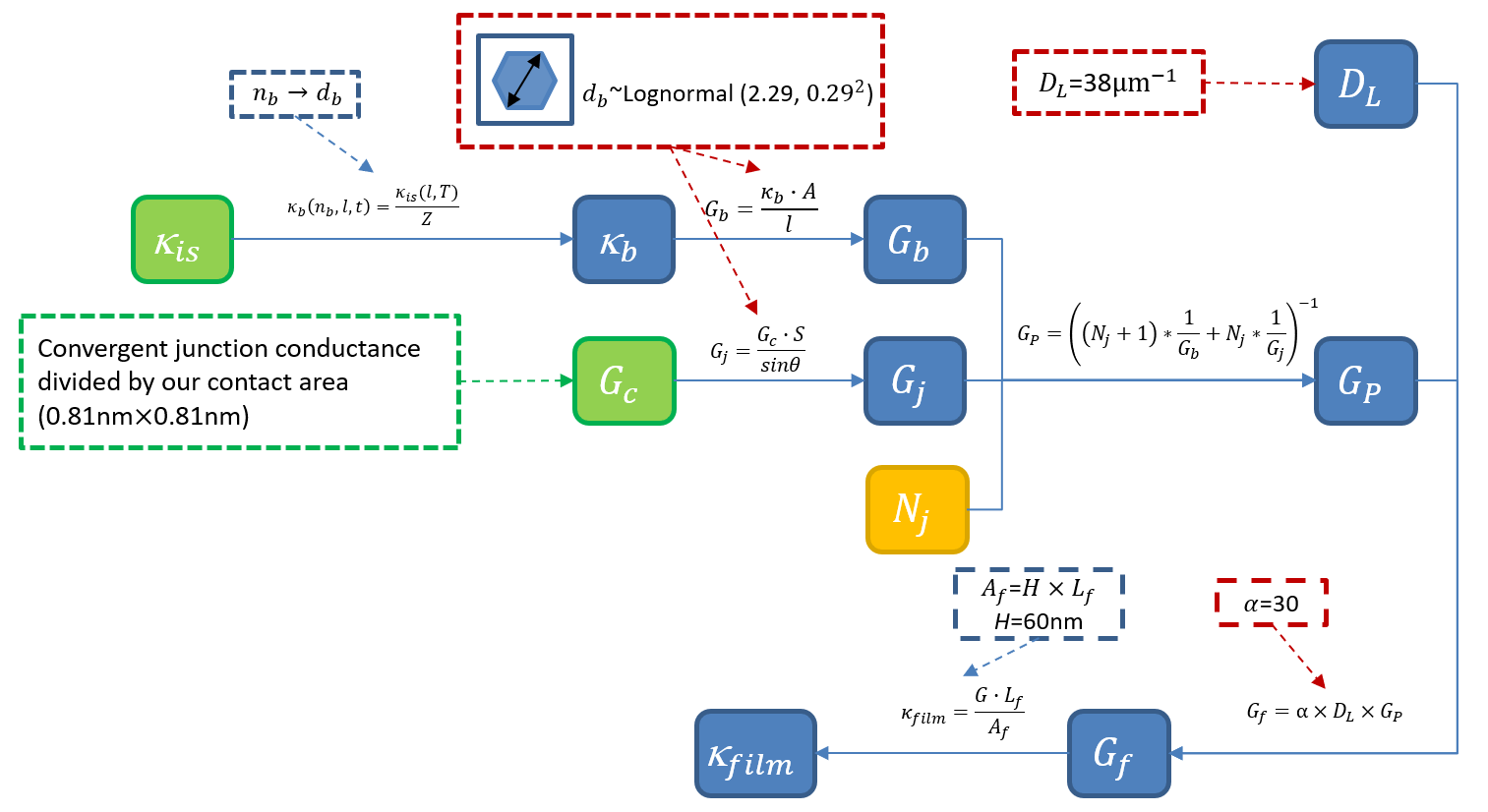


**Fig. S17** Flowchart for the calculation of film thermal conductivity. Red boxes are from experimental measurement of literature; green boxes are from our MD simulation; yellow boxes are from our Monte Carlo simulation.

The of a prinstine CNT film (${}_{film}$) using our simulated values increases with SWCNT length and overlaps with the results (Fig. S18) by Wang et. al.^[13]^ This increase in ${}_{film}$ arises from the SWCNT scaling of the intra-tube value with SWCNT length (Fig. 3d) and the reduced number of thermally resistive SWCNT junctions along each heat conduction path as SWCNT length increases. Fig S18 depicts that the MAPbI_3_ coating decreases the ${}_{film}$, mirroring the trend in our experiments. It is thus evident that the decrease of the *intra-tube* , from ${}_{SWCNT}$ to ${}_{SWCNT-MAPbI3}$, overwhelms the increase in the inter-tube junction conductance ${(G}_{j}),$ resulting in a smaller ${}_{film}$. This effect is particularly significant for films with longer SWCNTs.


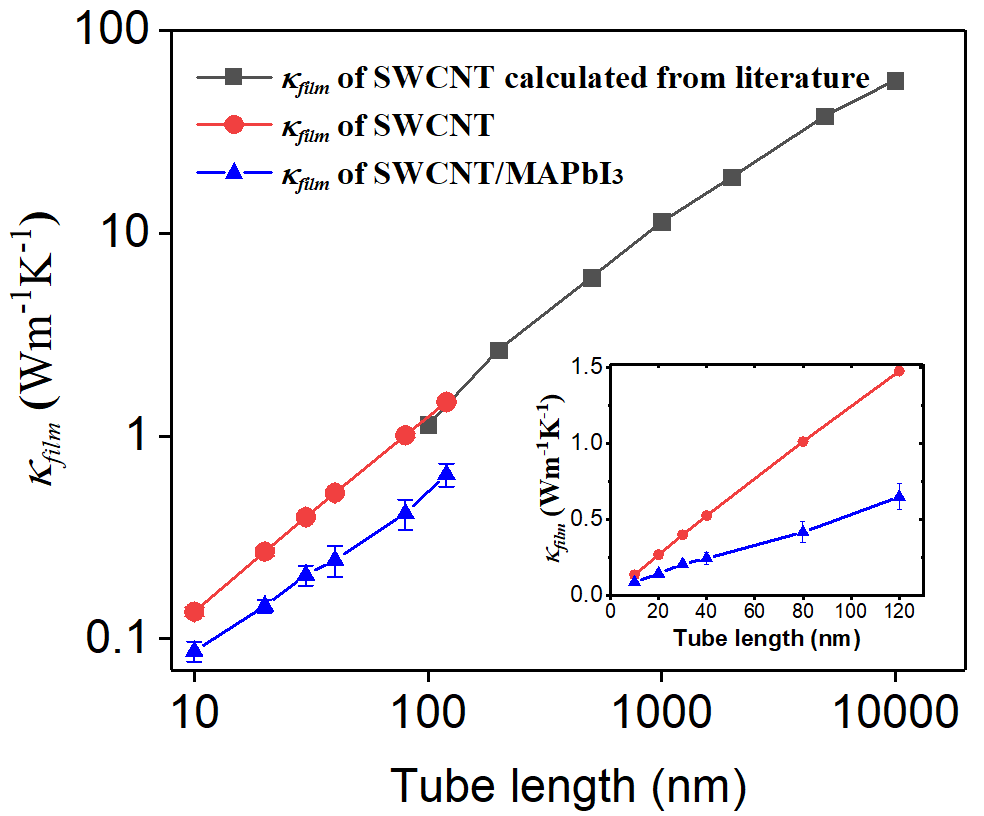


**Fig. S18** Thermal conductivity of pristine SWCNT and SWCNT/MAPbI_3_ films calculated using the statistical model.^[13]^ The black line is calculated by dividing the experimental sheet thermal conductance by its film thickness.^[13, 15]^ Our calculated thermal conductivity of the pristine SWCNT film using our simulated values (red) overlapped with the results (black) derived using the geometrical parameters from Wang et. al. The inset shows ${}_{film}$ in linear scale.


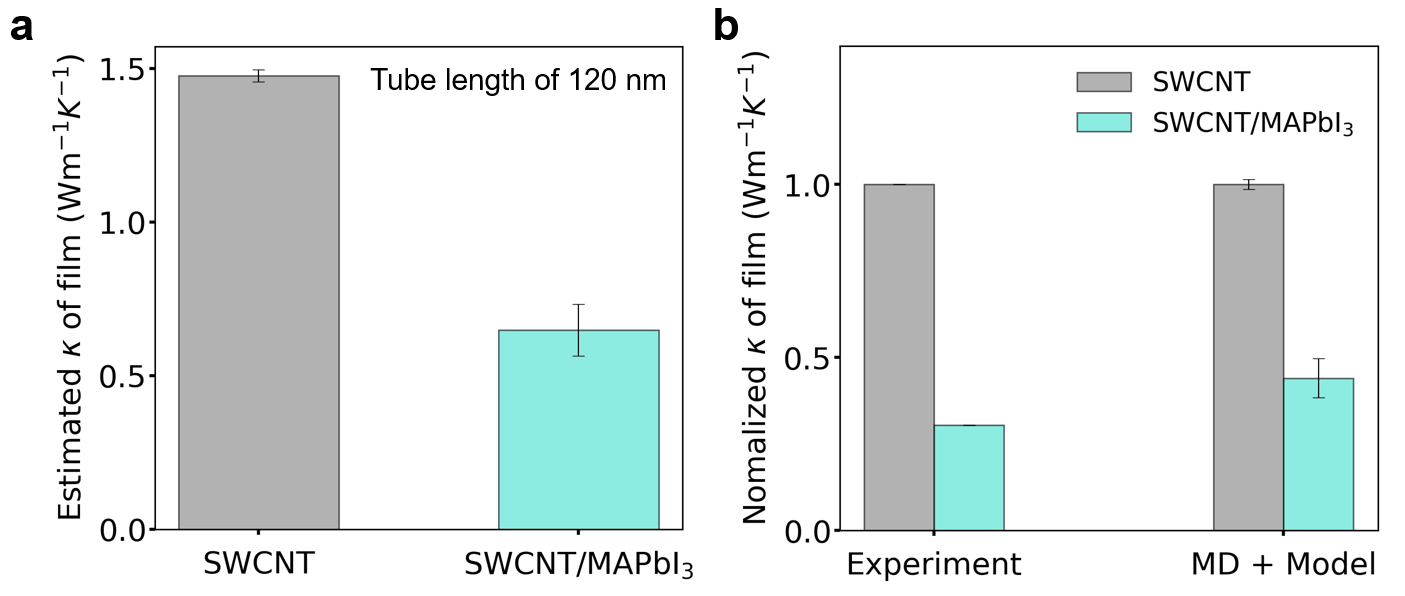


**Fig. S19** a) The estimated of a SWCNT film and SWCNT/MAPbI_3_ film with a tube length of 120 nm using MD simulation combined with the model from Ref.^[13]^. b) A comparison of the normalized of the SWCNT and SWCNT/MAPbI_3_ films using values from experiment and (a). Experiments and simulations show a similar decreasing trend after coating.


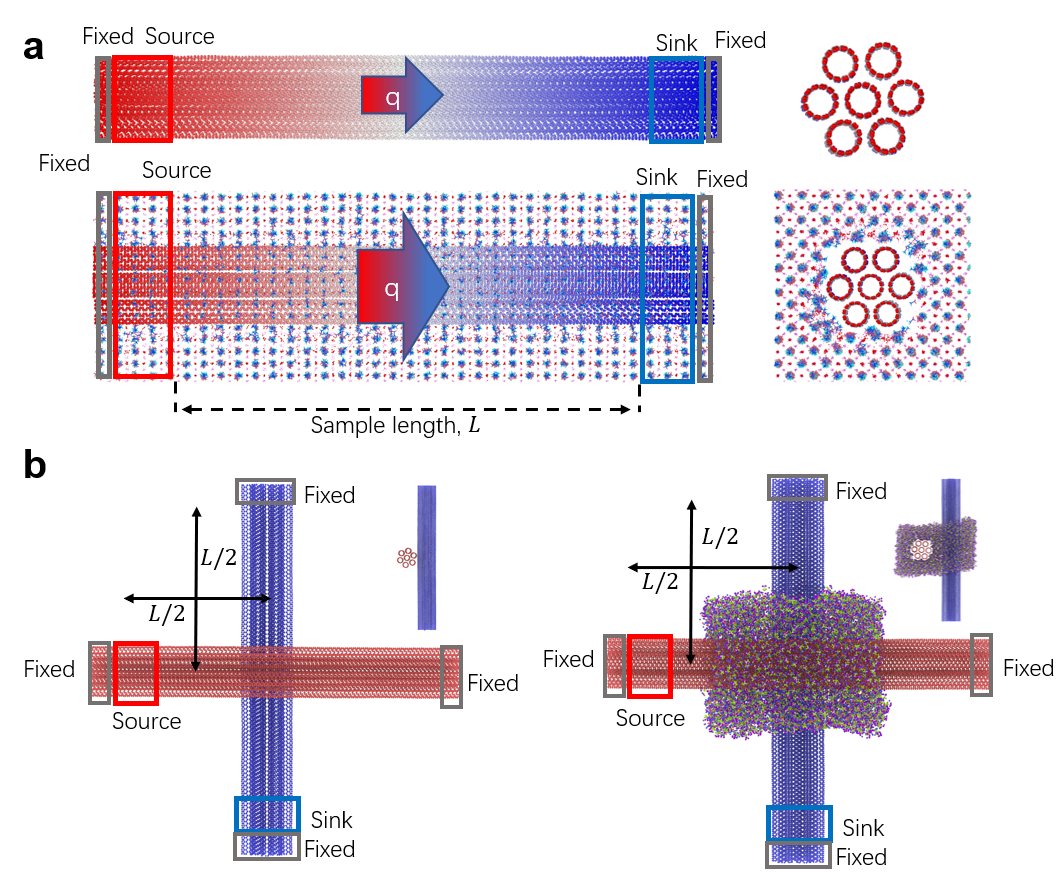


**Fig. S20** Schematics of the uncoated and MAPbI_3_-coated a) seven-tube bundle of SWCNT, b) junction of two seven-tube bundles of SWCNT/MAPbI_3_.


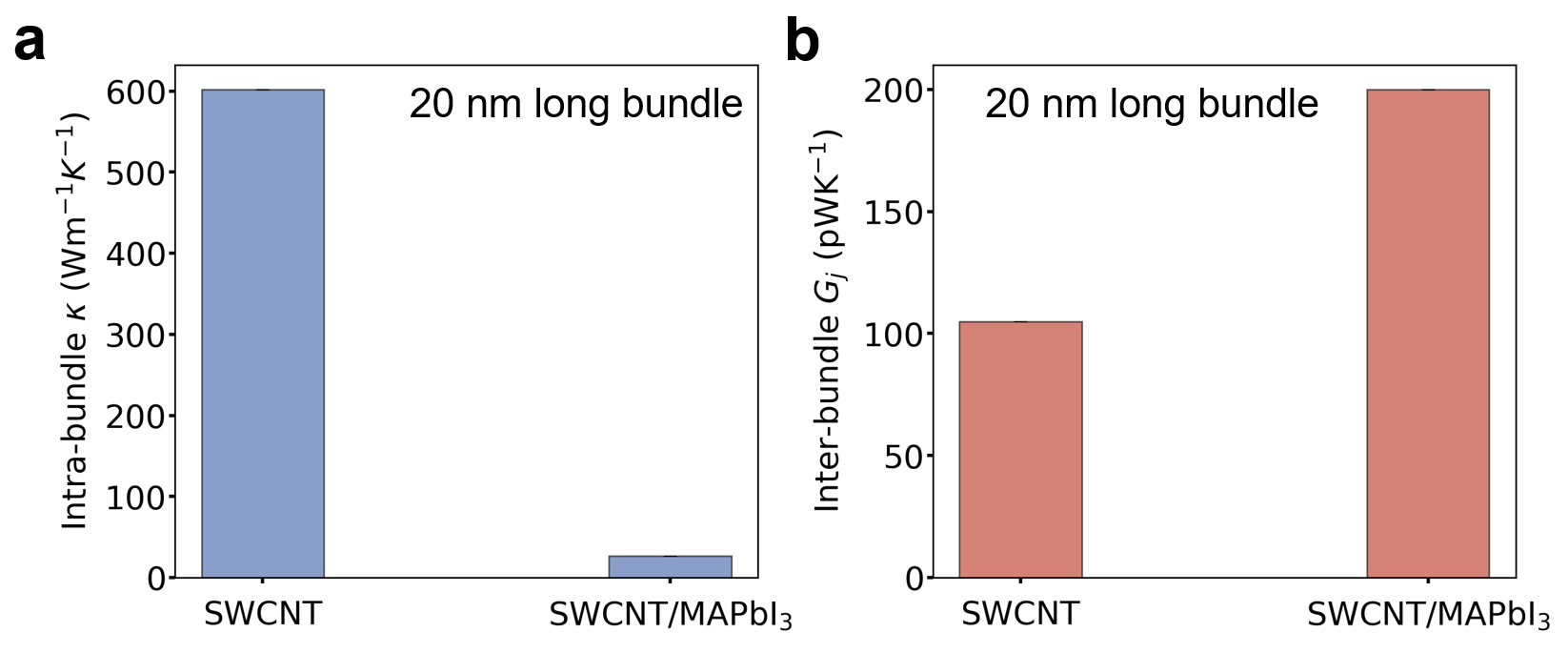


**Fig. S21** a) Intra-bundle of the 20 nm long seven-tube bundle of SWCNT and SWCNT/MAPbI_3_. b) Inter-bundle $G_{j}$ of the 20 nm long seven-tube bundle of SWCNT and SWCNT/MAPbI_3_.

**Supplementary note 3**

**A. Phonon density of states**

Phonon density of states (pDOS) of a SWCNT was calculated using the Fourier-transform of the velocity autocorrelation function (VACF),^[16]^

$\mathrm{pDOS}\left( \omega\right)= \int\left\langle\sum_{i=1}^{N} v_{i}\left( 0 \right)\cdot v_{i}\left( t \right) \right\rangle e^{-2\pi\cdot i\omega t}dt$ (S4)

where $\omega$ is the angular frequency, $N$ is the number of atoms, $v_{i}\left( t \right)$ is the velocity of the *i-*th atom at time *t*. After equilibrating for two nanoseconds under an NVE ensemble, the velocity was recorded every 16 steps (i.e., eight femtoseconds). The sampling duration was chosen as $2^{17}$ and $2^{19}$ steps to obtain a frequency resolution of 0.49 THz and 0.00383 THz, respectively.

**B. Phonon participation ratio**

The phonon participation ratio (PPR) was used to evaluate phonon localization,^[17]^

$PPR= \frac{1}{N}\frac{\left( \sum_{i} {DOS}_{i}\left( \omega\right)^{2} \right)^{2}}{\sum_{i} {DOS}_{i}\left( \omega\right)^{4}}$ (S5)

where ${DOS}_{i}(\omega)$ is the local density of states of atom $i$ obtained by calculating the single-particle VACF.^[18]^

**C. Spectral energy density**

The spectral energy density (SED) method was used to extract the anharmonic properties of SWCNT phonons.^[19]^ The expression of SED is defined as

$\Phi\left( k,\omega\right)=\frac{m}{4\pi n\tau_{0}}\sum_{\gamma} \sum_{b=1}^{B} \left| \int_{0}^{\tau_{0}} \sum_{l=1}^{n} u_{\gamma}\left( l,b,t \right)\times\exp\left( ik\cdot r_{l}-i\omega t \right)dt \right|^{2}$ (S6)

where $m$ is the mass of atoms, $n$ is the number of unit cells, $\tau_{0}$ is the total simulation time, $b$ is the atom index in each unit cell, $l$ is the index of each unit cell, $r_{l}$ is the equilibrium position of each unit cell, $u_{\gamma}$ is the atom velocity in $\gamma$ direction, $k$ is the wavevector. A phonon relaxation time is obtained by fitting the SED peaks as Lorentzian function ($\Phi$),

$\Phi\left( k,\omega\right)= \frac{I}{1+\left[ \frac{\left( \omega-\omega_{c} \right)}{\Gamma} \right]^{2}}$ (S7)

where $\omega_{c}$ is the frequency at peak center, $\Gamma$ is the half-width at half-maximum. The phonon relaxation time $\tau$ can then be defined as,

$\tau=\frac{1}{2\Gamma}$ (S8)

In our calculation, a SWCNT with 50 unit-cells (12.3 nm) was equilibrated in an NVE ensemble before data sampling. The atom trajectory was recorded every 8 fs for a total of 800 ps under an NVE ensemble at 300 K.

**D. Equilibrium molecular dynamics**

The thermal conductivity of MAPbI_3_ was calculated using NEMD and Green Kubo (GK) equilibrium molecular dynamic (EMD) simulation. In the GK approach, the thermal conductivity can be calculated by integrating the heat flux autocorrelation function,

${}_{x,y,z}= \frac{1}{k_{B}VT^{2}}\int_{0}^{\infty} \left\langle J_{x,y,z}(t)\cdot J_{x,y,z}(t) \right\rangle dt$ (S9)

where *T* and *V* are the temperature and volume of the system, $k_{B}$ is the Boltzmann constant, $J_{x,y,z}$ is the heat flux in three directions.

**
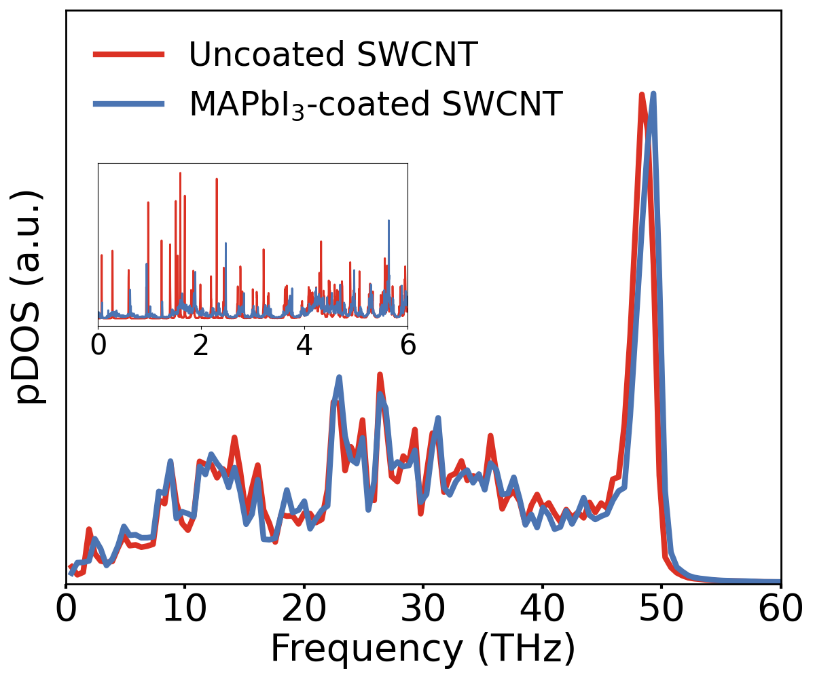
**

**Fig. S22** Phonon density of states of the uncoated and MAPbI_3_-coated SWCNT.

**Supplementary note 4**

The phonon dispersion of an uncoated SWCNT and MAPbI_3_-coated SWCNT is calculated using a five-unit-cell (120 atoms) supercell to match the lattice constant of MAPbI_3_. We identified these modes in Figure 4(b) using their eigenvectors (Figure S23). To clarify Figure 4(b), for example, the lowest branch that starts as a LA mode at the *Γ* point experiences two avoided crossings. This LA mode hybridizes to a TW and then a TA as a function of increasing wavevector. In addition, the degeneracy of the TA branches in the uncoated SWCNT was removed after coating.

Another critical component of the modal phonon contribution to thermal conductivity is its $\tau$. Here, the $\tau$ values were calculated using SED and plotted in Figure 4(d). Following the common practice of using the unchanged dispersion (uncoated D), the values of the blue $\tau$ points (SWCNT/MAPbI_3_), are smaller than the values of the red $\tau$ points (uncoated SWCNT), especially for the low-frequency phonons. The average $\tau$ decreases by 33% from 37.6 ps to 25.1 ps. Also evident from Figure S24, some of the low-frequency phonons [i.e., transverse acoustic and optical (TA and TO) modes] are more severely affected than the others [i.e., the longitudinal acoustic and optical (LA and LO) and the twisting (TW) modes]. When the coated-SWCNT dispersion is used (coated D), the calculated $\tau$ values are further reduced, with the average $\tau$ dropping by a further 29% from 25.1 ps to 17.8 ps.


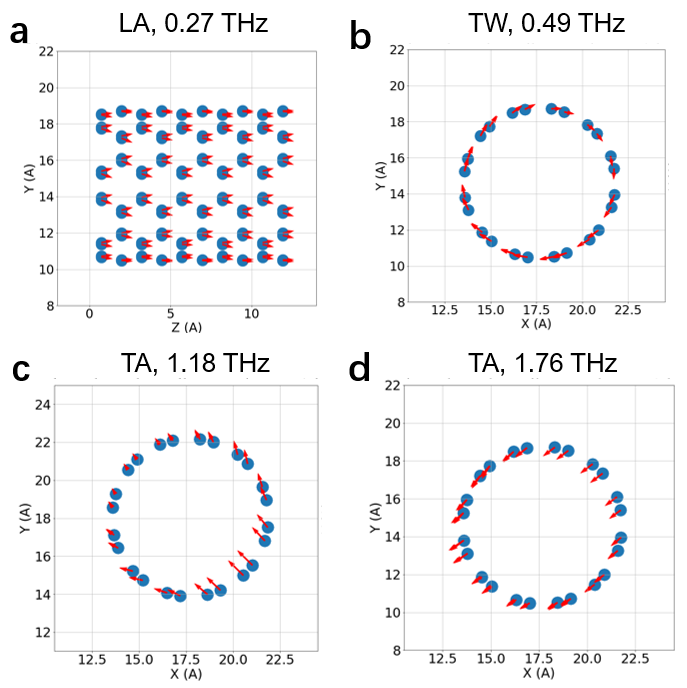


**Fig. S23** Eigenvectors of the acoustic phonons of a MAPbI_3_-coated SWCNT at the *Γ* point.


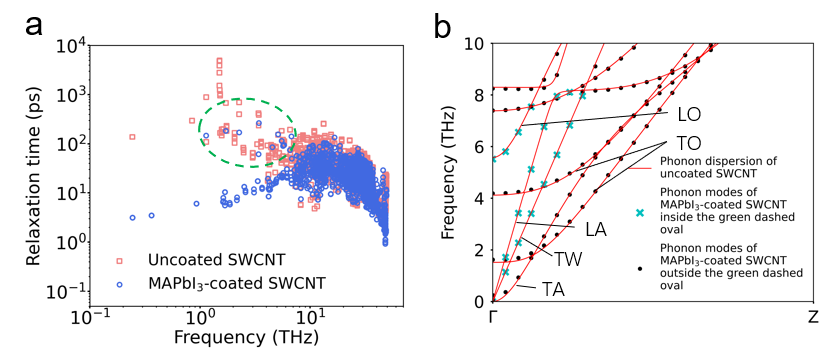


**Fig. S24** a) Phonon relaxation time of uncoated SWCNT and MAPbI_3_-coated SWCNT (uncoated D) obtained from SED b) Phonon modes below 10 THz. The cyan crosses correspond to the phonon modes of MAPbI_3_-coated SWCNT in the green dashed oval in (a).


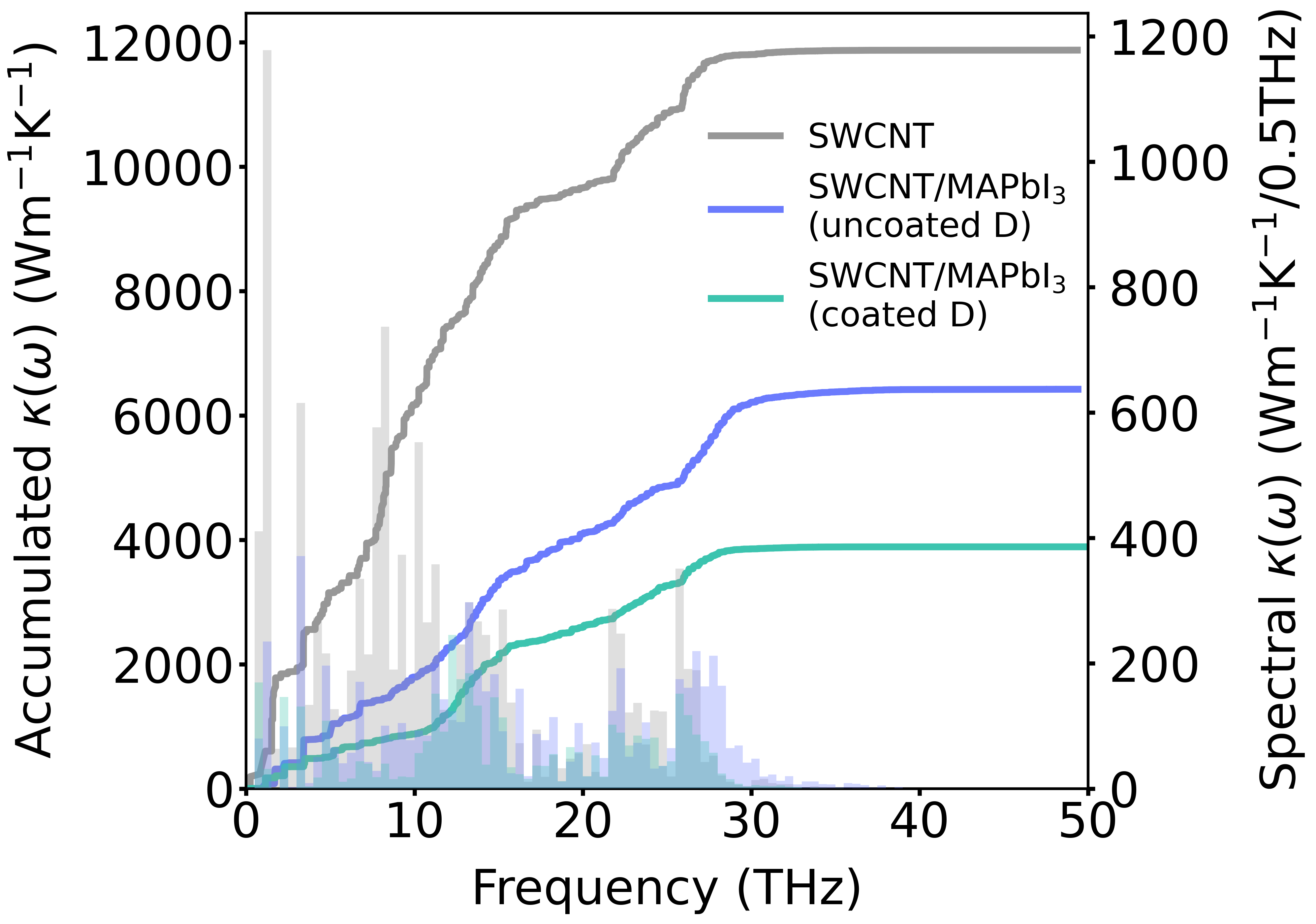


**Fig. S25** Spectral thermal conductivity and accumulated thermal conductivity of SWCNT, SWCNT/ MAPbI_3_ with the phonon dispersion (D) of an uncoated and coated SWCNT, respectively.

**Supplementary note 5**

The degree of coating is defined as the percentage of the coated length ($L_{coating}$) over the total SWCNT length (*L*), i.e., $\frac{L_{coating}}{L}x 100 \%$. The thermal conductivity of partially coated SWCNTs are calculated using NEMD following the same procedure as in Supplementary note 2. We supplement our study by calculating the thermal conductivity using the Boltzmann transport equation (BTE) under the relaxation time approximation. Such an approach is postulated to capture all the possible phonon modes, even with a short SWCNT.^[19, 20]^ Details of BTE calculations are provided at the end of this section. The $v_{g}$ and $\tau$ obtained using the “uncoated D” and “coated D” configurations are used to calculate the thermal conductivity.

Our result suggests that a partial coating of 20% can reduce the SWCNT thermal conductivity to 40%~60% of the uncoated SWCNT value under the SED+BTE approach. This reduction trend is similar to the NEMD result from an 80 nm long SWCNT shown in the same figure. The drop in thermal conductivity using the “coated D” configuration is more significant than that in the “uncoated D” configuration, as both the phonon group velocity and relaxation time of the former are lower. The thermal conductivity reduction is less in the NEMD than in the SED+BTE result due to the absence of longer wavelength phonons in the short SWCNTs in the NEMD approach. To test this hypothesis, the thermal conductivity of two longer SWCNTs with 20% coating was calculated and plotted as the empty solid triangle (250 nm) and dashed triangle (500 nm) in Figure S26(b). With longer SWCNTs, the reduction at the same coating degree becomes larger, confirming our postulation. We, thus, propose that for an ultralong MAPbI_3_-SWCNT, the converged thermal conductivity would approach the prediction by the SED+BTE approach in Figure S26(b).

The breakdown of the different phonon branches contributing to the SWCNT thermal conductivity is shown in Figures S26(c) and (d). In the fully-coated SWCNT, the contribution from the LA, TA, TW, and OPT modes are reduced by 66% (74%), 84% (98%), 46% (92%), and 38% (55%) from the uncoated SWCNT, resulting in a reduced total thermal conductivity by about 40% (60%) when the “uncoated D” (“coated D”) configuration is used. When the coating degree is more than 20%, the thermal conductivity contribution from the LA, TA, and TW modes reduces to as low as that of a fully-coated SWCNT. For the OPT modes, its contribution oscillates around the saturated value. The nature of this oscillation is currently unknown but similar to a prior report that studied argon-coated SWCNT.^[21]^


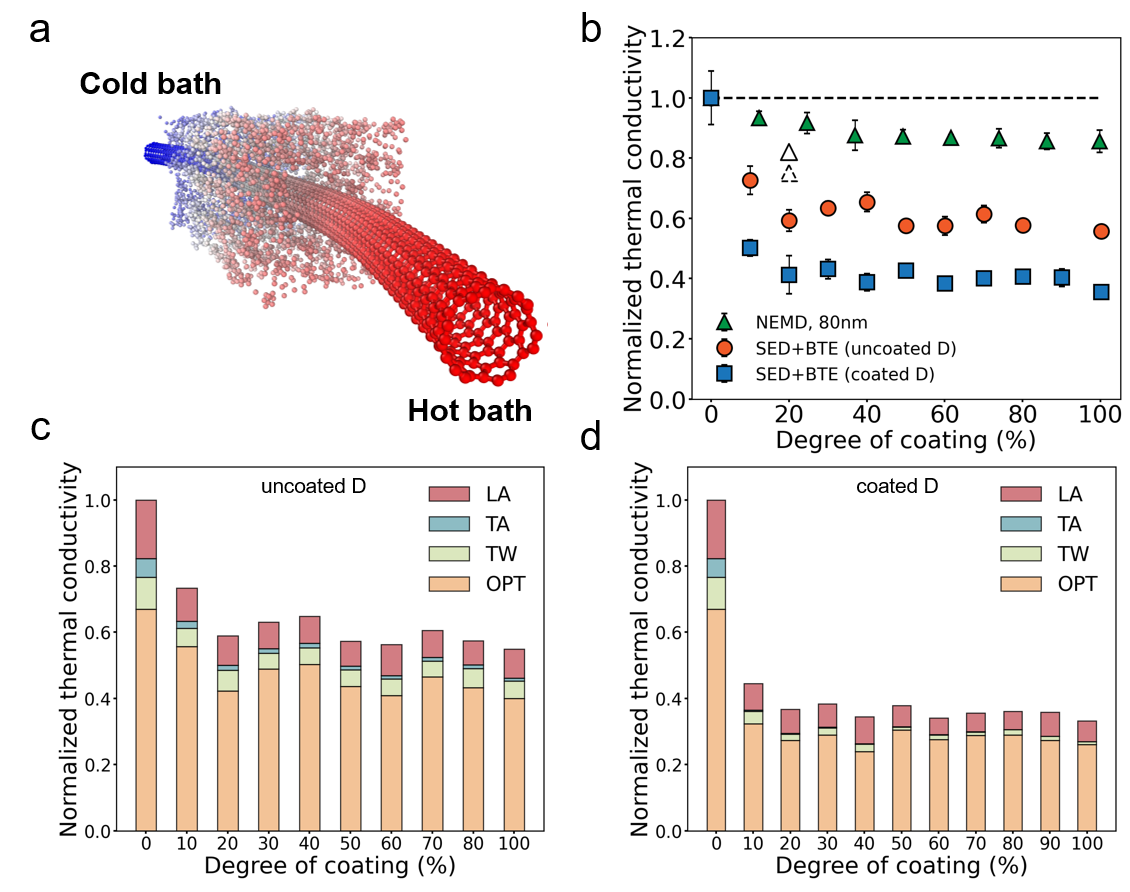


**Fig. S26** **a** Schematic of a partially coated MAPbI_3_-SWCNT. **b** Normalized thermal conductivity of the partially coated MAPbI_3_-SWCNT from NEMD and SED+BTE. The empty solid and dashed triangles are the NEMD thermal conductivity values using a SWCNT with a length of 250 nm and 500 nm, respectively. Contribution from the different phonon branches in a partially coated MAPbI_3_-SWCNT using **c** uncoated SWCNT dispersion (“uncoated D”) and **d** MAPbI_3_-coated SWCNT dispersion (“coated D”).

**Calculation of *_CNT_* using Boltzmann transport equation**

Employing the Boltzmann transport equation (BTE) under the relaxation time approximation, the thermal conductivity of SWCNT (${}_{CNT}$) can be calculated using Eq. (S10).^[19]^

${}_{CNT}= \sum_{q} \sum_{\nu} c_{ph}v_{g}^{2}\tau$ (S10)

In Eq. (S10), $c_{ph}$ is the phonon specific heat, $v_{g}$ is the group velocity, $\tau$ is the relaxation time, $q$ and $\nu$ are used to represent the different phonon wavevectors and frequencies. Considering the classical nature of MD simulation, we use the classical limit of the specific heat $c_{ph}={k_{B}}/V$, where $k_{B}$ is the Boltzmann constant and $V$ is the SWCNT volume.


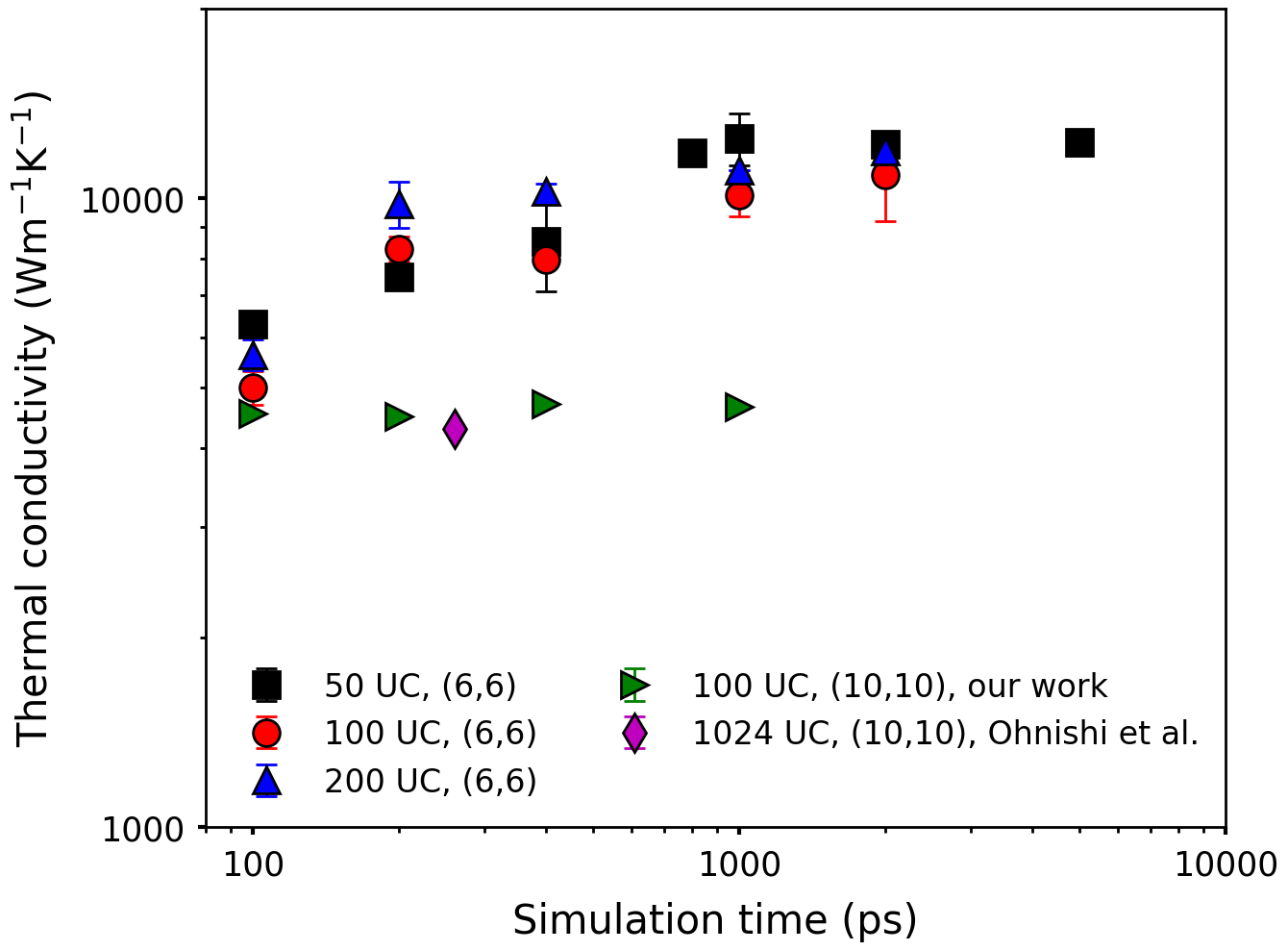


**Fig. S27** Thermal conductivity of uncoated SWCNT calculated from SED+BTE.

A converged relaxation time determined using the SED method requires a long simulation. The convergence test for the BTE-predicted ${}_{CNT}$ is shown in Figure S27. It can be seen that for a 50-unit-cell SWCNT, integrating beyond 800 ps does not change the BTE-predicted ${}_{CNT}$. Also, no clear length dependence can be seen for the thermal conductivity of 50UC, 100UC, and 200UC long SWCNT, which is consistent with Ohnishi et al.^[20]^ Therefore, we expect that results from a SWCNT of 50-unit-cell and a simulation time of 800 ps is sufficient to reproduce results from longer tubes. We validated our choice by calculating and comparing the thermal conductivity of a (10, 10) SWCNT with results from Ohnishi et al., which has a smaller than a (6,6) SWCNT.

**Supplementary note 6**

The thermal conductivity of the three MAPbI_3_ structures shown in Figure 4(g) are calculated using NEMD following the same procedure as in Supplementary note 2. The thermal conductivity contribution from the organic molecules (MA) and inorganic cage (PbI) inside the MAPbI_3_ solid are calculated from the Green Kubo method and shown in Figure S28. For the Cuboid case, the contribution from the MA and PbI agree reasonably well with the existing literature.^[9]^ The thermal conductivity contributed by the MA and PbI decreases by 23.5% and 24% in the Tube case and a further 23% and 10.5% in the Tube-SWCNT case. This result shows that the amorphization of the MAPbI_3_ and the coupling to the SWCNT surface reduce the MAPbI_3_ thermal conductivity. The initial amorphization seems to decrease the contribution from both the MA and PbI equally, while the insertion of the SWCNT decreases the thermal conductivity contribution from the MA more significantly.


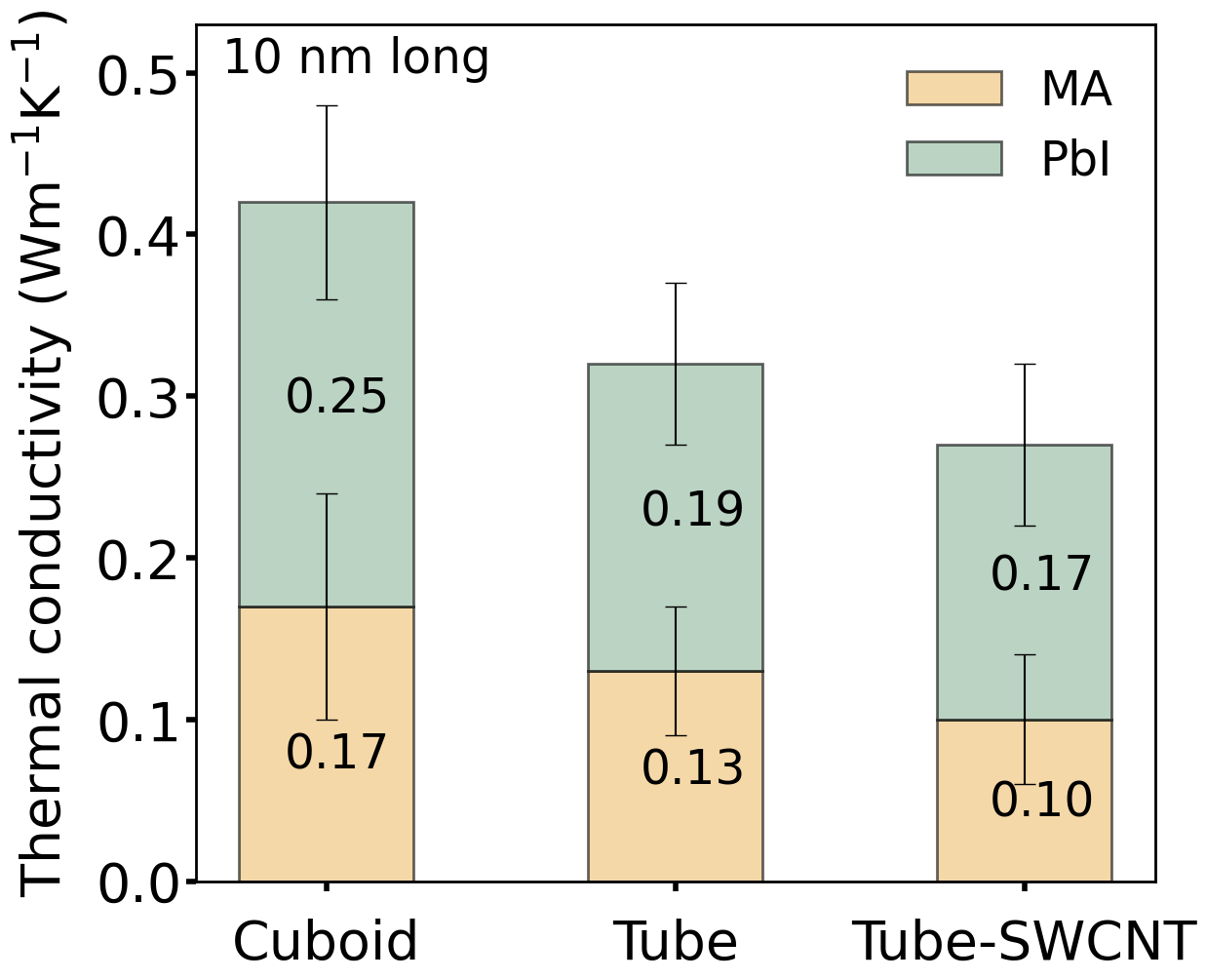


**Fig. S28** Contribution of the organic and inorganic part to the thermal conductivity of MAPbI_3_ for a 10 nm long system. The uncertainty bars are calculated from 400 independent runs (see Figure S29).

**Green Kubo EMD**


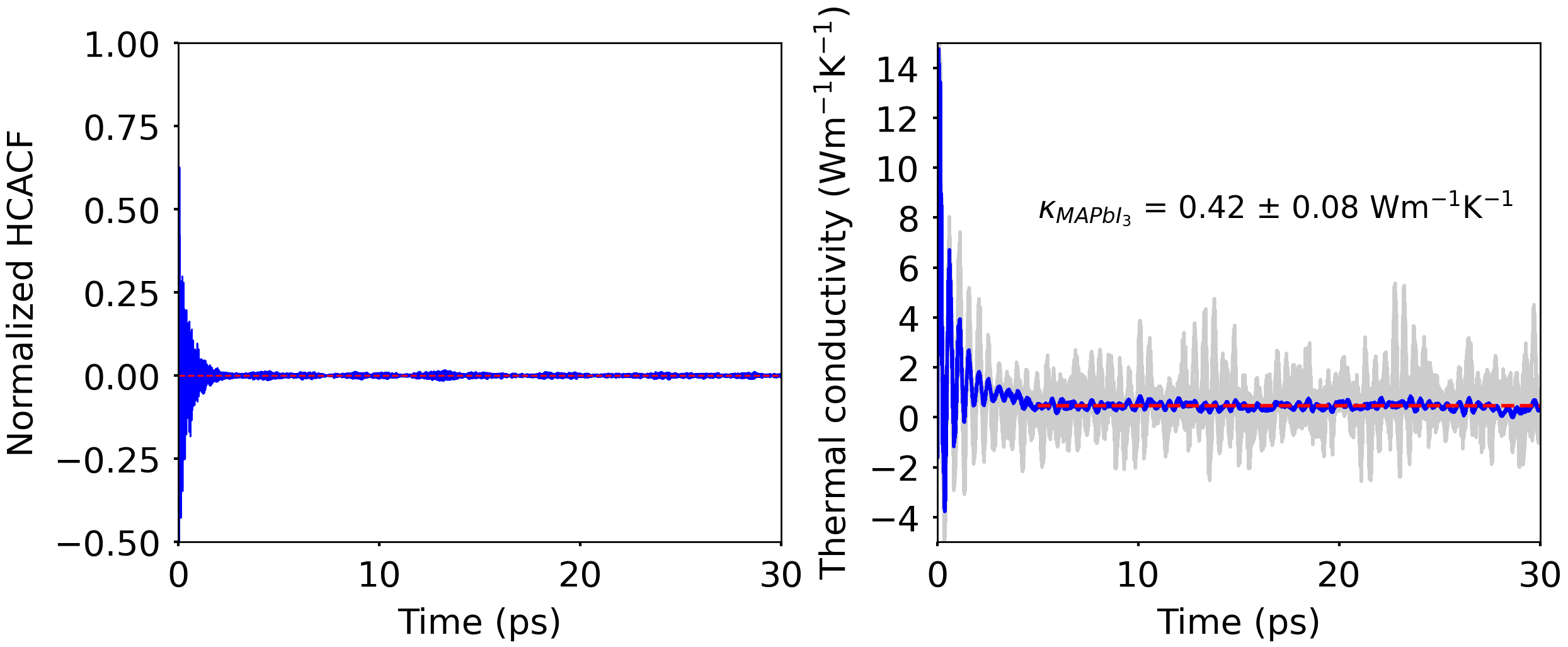


**Fig. S29** Heat flux autocorrelation function and thermal conductivity calculation of MAPbI_3_.

From an atomistic perspective, the variations in the Pb-I bond length and the Pb-I-Pb angle suggest that the inorganic lattice is distorted by the hollow tube and the inserted SWCNT (Table S3). However, the bond length and angle in MA are relatively unaffected, except for the N-H bond. A slight stretch of the N-H bond from its equilibrium length possibly arises from the strong coupling with the PbI cage through strong electrostatic interactions and hydrogen bonds, which can strengthen the anharmonicity and reduce thermal conductivity.^[22, 23]^ Nonetheless, the structural difference in the MA molecules between the Tube and the Tube-SWCNT case is minimal. In Figure S30(a), the calculated mean square displacement (MSD) of the atoms in MA is lower in the Tube-SWCNT case, which suggests a suppression of the vibrations. This decrease in anharmonicity seems to contradict the reduced MA contribution to the thermal conductivity in Figure S28. This contradiction is explored in the next paragraph. In contrast, the MSD of the atoms in the PbI in Figure S30(b) increases in the Tube case due to an increase in the number of dangling Pb-I bonds (Figure S31) but decreases in the presence of the SWCNT due to interactions with the SWCNT. Although a smaller MSD implies weaker anharmonicity resulting in less phonon-phonon scattering,^[24]^ the presence of the SWCNT also introduces interfacial scattering, which can decrease the phonon relaxation time.^[25]^ Hence, there are two competing mechanisms in the Tube-SWCNT case that influence the thermal conductivity of the MAPbI_3_.

Table S3. Bond lengths and angles of MAPbI_3_ calculated by averaging over a one-million-step MD run in an NVE ensemble.

| Bond length (A) | Cuboid | Tube | Tube-SWCNT |
| --- | --- | --- | --- |
| C-N | 1.490 ± 0.003 | 1.490 ± 0.003 | 1.491 ± 0.003 |
| N-H | 1.039 ± 0.001 | 1.042 ± 0.002 | 1.042 ± 0.002 |
| C-H | 1.093 ± 0.002 | 1.093 ± 0.002 | 1.093 ± 0.002 |
| Pb-I | 3.165 ± 0.156 | 3.108 ± 0.281 | 3.115 ± 0.286 |
| Angle (°) | Cuboid | Tube | Tube-SWCNT |
| H-C-N | 109.378 ± 0.009 | 109.377 ± 0.010 | 109.379 ± 0.010 |
| H-N-C | 109.346 ± 0.011 | 109.336 ± 0.013 | 109.338 ± 0.013 |
| Pb-I-Pb | 168.502 ± 5.680 | 160.866 ± 13.840 | 160.986 ± 14.268 |


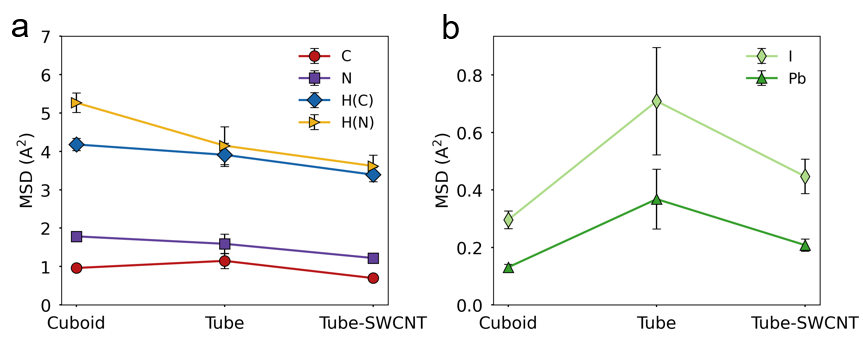


**Fig. S30** a) Mean square displacement of the atoms in the MA molecules. b) Mean square displacement of the atoms in the PbI cage.

To investigate the above contradiction in the MA contribution, we calculated the phonon participation ratio (PPR) in the MAPbI_3_ within the Lennard Jones interaction cutoff distance from the SWCNT surface (Fig. S32-33). These PPR are weighted by their corresponding pDOS to suppress the baseline noise. From Figure 4(h), the PPR_Cuboid,MA_ > PPR_Tube,MA_ > PPR_Tube-SWCNT,MA_ for frequencies below 25 THz, signifying an increase in the phonon localization near the hollow tube surface, that can be responsible for the decrease in thermal conductivity contribution from the MA. This PPR trend mirrors the calculated MSD in Figure S30(a). Such a positive correlation between the PPR and MSD was also previously found in an encapsulated graphene system.^[26]^

**Calculation of bond length and angle**


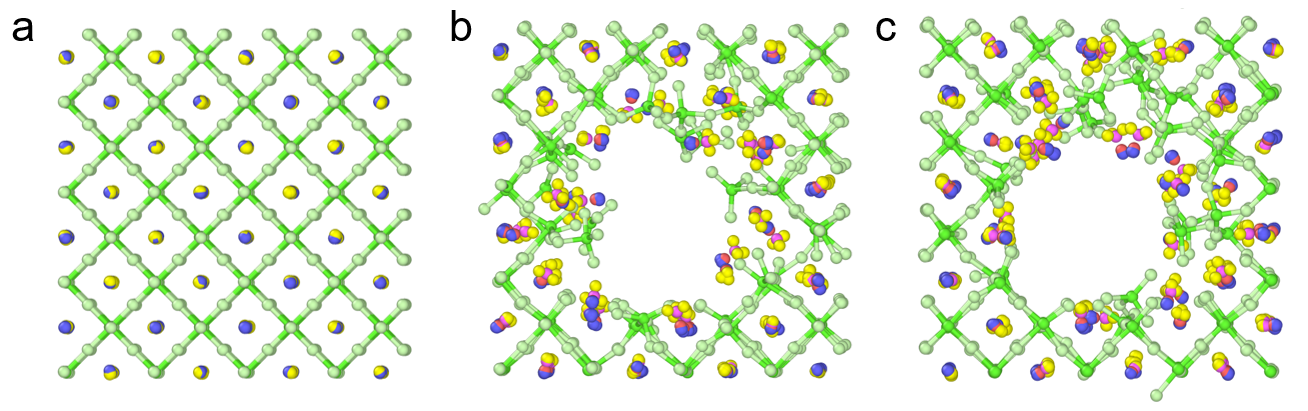


**Fig. S31** Average position (sampled from a one-million-step MD run) of the MAPbI_3_ in **a** Cuboid **b** Tube **c** Tube-SWCNT [yellow: H(N), purple: H(N), red: C, blue: H(C), dark green: Pb, light green: I]

**Phonon participation ratio**


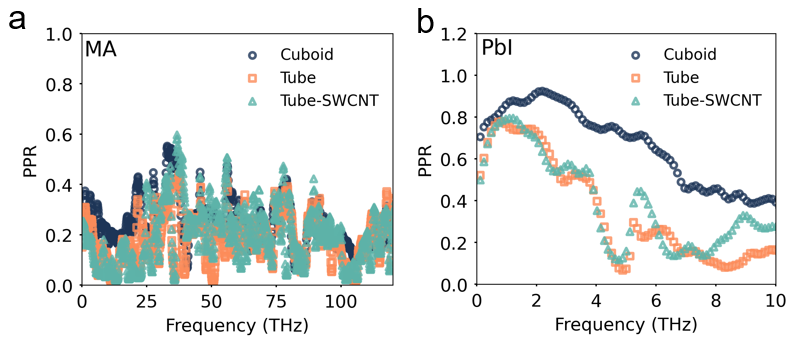


**Fig. S32** Phonon participation ratio of the MA and PbI in the Cuboid, Tube, and Tube-SWCNT cases.


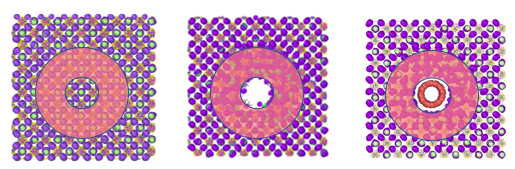


**Fig. S33** Phonon participation ratio calculated using the atoms in the red region that is within the LJ cutoff distance from the SWCNT.

**Effect of the strength of LJ potential between SWCNT and MAPbI_3_**

To test how the LJ interaction strength affects the thermal transport, the potential well depth *ε* in the LJ potential between SWCNT and MAPbI_3_ is scaled between 0.1 to 20 times of the original value. When the scaling factor is larger than 20, the MAPbI_3_ structure collapses, signifying it is inappropriate to go beyond 20. The result in Figure S34 shows that the intra-tube decreases while the inter-tube $G_{j}$ increases as the scaling factor increases. This trend arises as the stronger coupling induces more interfacial phonon scattering and suppresses thermal transport in the intra-tube direction but facilitates the thermal transport in the inter-tube direction. However, the estimated of SWCNT/MAPbI_3_ film remains smaller than the of SWCNT film regardless of the *ε* value. Therefore, varying the interaction strength between SWCNT and MAPbI_3_ does not change our conclusion that the MAPbI_3_ coating reduces the overall of a SWCNT film.


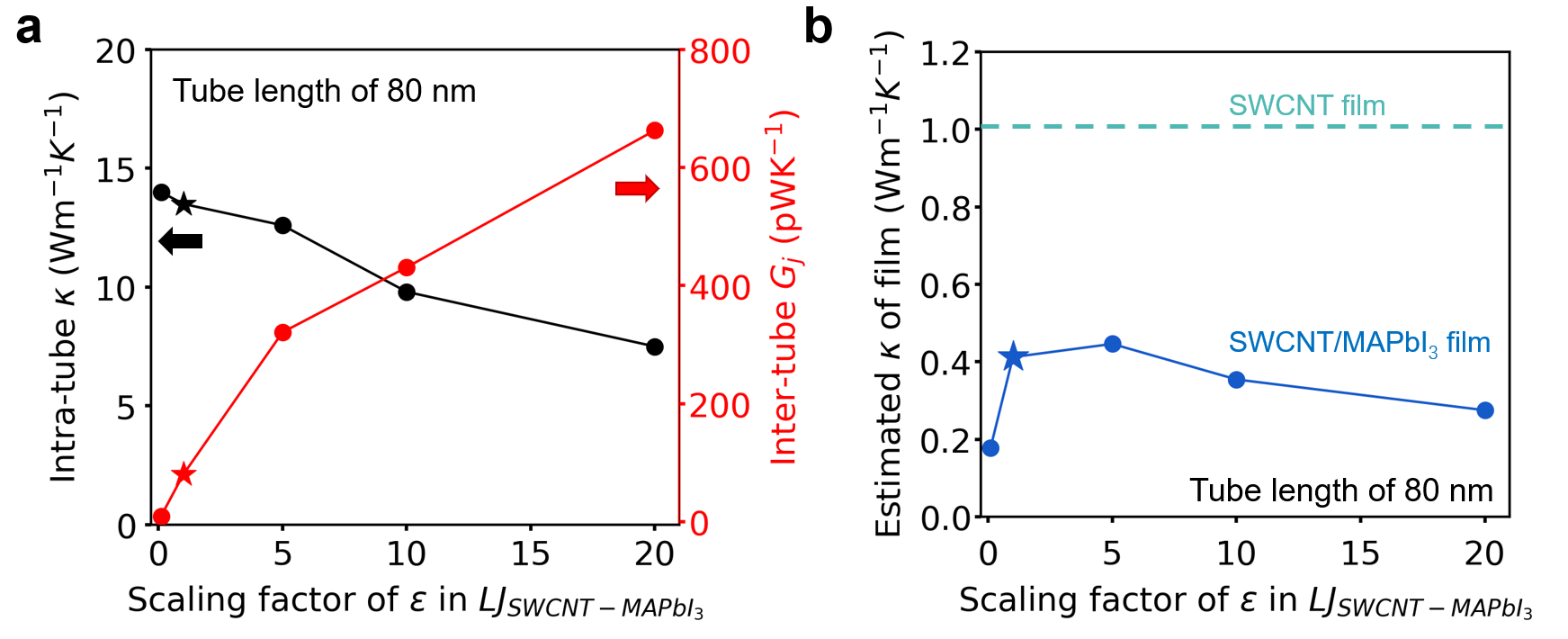


**Fig. S34** a) The intra-tube and inter-tube $G_{j}$ of an 80 nm long SWCNT/MAPbI_3_ with different scaling factors of parameter *ε* in the LJ potential between SWCNT and MAPbI_3_. b) The resulting of SWCNT/MAPbI_3_ film estimated using data from (a) as inputs to the ${}_{film}$ model.^[13]^ The values obtained using the original LJ potential are indicated as stars.

**References**

[1] M. A. Haque, T. Zhu, L. H. Hernandez, R. Tounesi, C. Combe, B. Davaasuren, A.-H. Emwas, F. P. García de Arquer, E. H. Sargent, D. Baran, Electrical tunability of inorganic tin perovskites enabled by organic modifiers, *Cell. Rep. Phys. Sci.* **2023**, *4*, 101703.

[2] L. Weston, H. Tailor, K. Krishnaswamy, L. Bjaalie, C. G. Van de Walle, Accurate and efficient band-offset calculations from density functional theory, *Comput. Mater. Sci.* **2018**, *151*, 174.

[3] G. Kresse, J. Furthmüller, Efficiency of ab-initio total energy calculations for metals and semiconductors using a plane-wave basis set, *Comput. Mater. Sci.* **1996**, *6*, 15.

[4] V. Wang, N. Xu, J.-C. Liu, G. Tang, W.-T. Geng, VASPKIT: A user-friendly interface facilitating high-throughput computing and analysis using VASP code, *Comput. Phys. Commun.* **2021**, *267*, 108033.

[5] L. Lindsay, D. A. Broido, Optimized Tersoff and Brenner empirical potential parameters for lattice dynamics and phonon thermal transport in carbon nanotubes and graphene, *Phys. Rev. B* **2010**, *81*, 205441.

[6] L. Hu, A. J. H. McGaughey, Thermal conductance of the junction between single-walled carbon nanotubes, *Appl. Phys. Lett.* **2014**, *105*, 193104.

[7] A. Mattoni, A. Filippetti, M. I. Saba, P. Delugas, Methylammonium Rotational Dynamics in Lead Halide Perovskite by Classical Molecular Dynamics: The Role of Temperature, *J. Phys. Chem. C* **2015**, *119*, 17421.

[8] Y. Gao, W. Ning, X. Zhang, Y. Liu, Y. Zhou, D. Tang, The effective regulation of nanotwinning on the multichannel thermal transport in hybrid organic–inorganic halide perovskite, *Nano Energy* **2021**, *82*, 105747.

[9] A. Giri, Origins of pressure-induced enhancement in thermal conductivity of hybrid inorganic–organic perovskites, *Nanoscale* **2021**, *13*, 685.

[10] C. Caddeo, C. Melis, M. I. Saba, A. Filippetti, L. Colombo, A. Mattoni, Tuning the thermal conductivity of methylammonium lead halide by the molecular substructure, *Phys. Chem. Chem. Phys.* **2016**, *18*, 24318.

[11] M. P. Allen, M. P. Allen, D. J. Tildesley, D. J. Tildesley, T. ALLEN, *Computer Simulation of Liquids*. (Clarendon Press, 1989).

[12] A. P. Thompson, H. M. Aktulga, R. Berger, D. S. Bolintineanu, W. M. Brown, P. S. Crozier, P. J. in 't Veld, A. Kohlmeyer, S. G. Moore, T. D. Nguyen, R. Shan, M. J. Stevens, J. Tranchida, C. Trott, S. J. Plimpton, LAMMPS - a flexible simulation tool for particle-based materials modeling at the atomic, meso, and continuum scales, *Comput. Phys. Commun.* **2022**, *271*, 108171.

[13] P. Wang, Y. Feng, R. Xiang, T. Inoue, A. Anisimov, E. I. Kauppinen, S. Chiashi, S. Maruyama, Phenomenological model of thermal transport in carbon nanotube and hetero-nanotube films, *Nanotechnology* **2021**, *32*, 205708.

[14] E. Pop, D. Mann, Q. Wang, K. Goodson, H. Dai, Thermal Conductance of an Individual Single-Wall Carbon Nanotube above Room Temperature, *Nano Lett.* **2006**, *6*, 96.

[15] S. Yoshida, Y. Feng, C. Delacou, T. Inoue, R. Xiang, R. Kometani, S. Chiashi, E. I. Kauppinen, S. Maruyama, Morphology dependence of the thermal transport properties of single-walled carbon nanotube thin films, *Nanotechnology* **2017**, *28*, 185701.

[16] J. M. Dickey, A. Paskin, Computer Simulation of the Lattice Dynamics of Solids, *Phys. Rev.* **1969**, *188*, 1407.

[17] S. E. Burkov, B. E. C. Koltenbah, L. W. Bruch, Phonon localization in one-dimensional quasiperiodic chains, *Phys. Rev. B* **1996**, *53*, 14179.

[18] K. Xu, T. Liang, Z. Zhang, X. Cao, M. Han, N. Wei, J. Wu, Grain boundary and misorientation angle-dependent thermal transport in single-layer MoS2, *Nanoscale* **2022**, *14*, 1241.

[19] J. A. Thomas, J. E. Turney, R. M. Iutzi, C. H. Amon, A. J. H. McGaughey, Predicting phonon dispersion relations and lifetimes from the spectral energy density, *Phys. Rev. B* **2010**, *81*, 081411.

[20] M. Ohnishi, J. Shiomi, Strain-induced band modulation of thermal phonons in carbon nanotubes, *Phys. Rev. B* **2021**, *104*, 014306.

[21] C. F. Carlborg, J. Shiomi, S. Maruyama, Thermal boundary resistance between single-walled carbon nanotubes and surrounding matrices, *Phys. Rev. B* **2008**, *78*, 205406.

[22] M. Grechko, S. A. Bretschneider, L. Vietze, H. Kim, M. Bonn, Vibrational Coupling between Organic and Inorganic Sublattices of Hybrid Perovskites, *Angew. Chem. Int. Ed.* **2018**, *57*, 13657.

[23] J. S. Bechtel, R. Seshadri, A. Van der Ven, Energy Landscape of Molecular Motion in Cubic Methylammonium Lead Iodide from First-Principles, *J. Phys. Chem. C* **2016**, *120*, 12403.

[24] K. D. Parrish, A. Jain, J. M. Larkin, W. A. Saidi, A. J. H. McGaughey, Origins of thermal conductivity changes in strained crystals, *Phys. Rev. B* **2014**, *90*, 235201.

[25] Z.-Y. Ong, E. Pop, J. Shiomi, Reduction of phonon lifetimes and thermal conductivity of a carbon nanotube on amorphous silica, *Phys. Rev. B* **2011**, *84*, 165418.

[26] J. D. Thomsen, T. Gunst, S. S. Gregersen, L. Gammelgaard, B. S. Jessen, D. M. A. Mackenzie, K. Watanabe, T. Taniguchi, P. Bøggild, T. J. Booth, Suppression of intrinsic roughness in encapsulated graphene, *Phys. Rev. B* **2017**, *96*, 014101.
